# The seven deadly sins: measuring overvaluation of social media with the Plan-net 25 scale

**DOI:** 10.1186/s40359-025-02801-1

**Published:** 2025-05-27

**Authors:** Víctor Ciudad-Fernández, Alfredo Zarco-Alpuente, Tamara Escrivá-Martínez, Elena Gomis-Vicent, Begoña Espejo, Óscar Lecuona, José C. Perales, Olatz Lopez-Fernandez, Rosa Baños

**Affiliations:** 1 Department of Personality, Evaluation, and Psychological Treatments, University of Valencia, Valencia, 46010 Spain; 2 Polibienestar Institute, University of Valencia, Valencia, 46022 Spain; 3 Department of Basic Psychology, University of Valencia, Valencia, 46010 Spain; 4 CIBERObn Physiopathology of Obesity and Nutrition, Instituto de Salud Carlos III, Madrid, 28029 Spain; 5 Department of Behavioral Sciences Methodology, University of Valencia, Valencia, 46010 Spain; 6 Department of Psychobiology and Methodology, Complutense University of Madrid, Madrid, 28223 Spain; 7 Department of Experimental Psychology, Mind, Brain and Behavior Research Center (CIMCYC), University of Granada, Granada, 18071 Spain; 8 Department of Methodology of Behavioural Sciences, National University of Distance Education, Madrid, 28040 Spain; 9 Department of Personality, Assessment, and Clinical Psychology, Complutense University of Madrid, Madrid, 28223 Spain

**Keywords:** Problematic social media use, Validation, Psychometric properties, Scale development, Overvaluation of social media, Social media

## Abstract

**Background:**

Problematic social media use refers to the excessive and maladaptive use of social media platforms, which negatively affects personal, social, and professional functioning. Although linked to mental health issues such as depression, anxiety, and loneliness, the underlying mechanisms remain unclear. A potential contributing factor to Problematic Social Media Use (PSMU) is the overvaluation of the relative utility of social media, where individuals disproportionately overvalue social media for different utility domains (e.g., communication or emotional regulation). This study aimed to develop and validate the Plan-net 25 scale, which was designed to assess overvaluation of the relative utility of social media in adolescents.

**Methods:**

The study followed three phases. Initially, a Delphi panel of 14 experts evaluated items across different utility domains. A pilot study involving 17 adolescents was conducted, and cognitive interviews were subsequently used to refine the scale items. Finally, the scale was administered to a large sample of 2,477 adolescents aged 12–20 years in Spain, alongside assessments of depression, anxiety, loneliness, life satisfaction, self-esteem, and problematic social media use. The analyses included confirmatory factor analysis, Pearson correlation, and network analysis, all of which were conducted via R 4.3.2.

**Results:**

Confirmatory factor analysis supported the theoretical seven-factor structure, capturing the following overvaluation of the relative utility of social media domains: social interaction, meeting new people, emotional regulation, social acceptance, staying informed, self-expression, and boredom management. The scale demonstrated full measurement invariance across gender and age groups (early and late adolescence). Significant correlations were found between overvaluation of the relative utility of social media, problematic social media use, and mental health indicators, with the emotional regulation and entertainment overvaluation of the relative utility of social media domains showing the strongest associations with problematic social media use.

**Conclusions:**

The Plan-net-25 scale exhibited robust psychometric properties, suggesting that it is a promising tool for assessing overvaluation of the relative utility of social media during adolescence.

**Supplementary Information:**

The online version contains supplementary material available at 10.1186/s40359-025-02801-1.

## Introduction

PSMU has been defined as the excessive use of social media (SM) platforms with detrimental consequences for a user’s personal, professional or social functioning [1]. In association with decreased life satisfaction and self-esteem, PSMU is also correlated with increased depression, anxiety, stress and loneliness [2, 3]. Despite extensive research, specific mechanisms that initiate, maintain, and exacerbate PSMU remain unclear. For instance, psychological disorders such as depression or anxiety may not only result from PSMU but could also serve as underlying risk factors that predispose individuals to developing PSMU [4].

Furthermore, the literature has identified risk factors such as impulsivity and emotional regulation difficulties in both substance and behavioral addictions [5–8]. Additionally, decision-making-related factors, particularly the overvaluation of certain actions within specific contexts, have been proposed to play a relevant role in these disorders [9, 10]. Overvaluation has been extensively studied in drug addiction and gambling disorders [9–12] and could also be a potential unexplored mechanism for understanding PSMU.

When individuals overestimate the value of an action or its outcomes in a specific situation, it can lead to overselection [10]. Research in the field of drug addiction shows that individuals with this problem show a decision-making bias, as they overvalue drug-specific reinforcement [13]. Hence, they prefer performing the overvalued behavior over other alternatives, paving the way for greater disorder severity [14]. Moreover, such overvaluation is often accompanied by expectancy bias, wherein individuals hold inflated expectations of rewards that do not align with the actual value or satisfaction experienced when the reward is attained [15].

Overvaluation is a risk factor relevant not only to substance use and gambling disorders but also to other excessive behaviors [16]. For example, in compulsive buying disorder, both the act of buying and the acquired objects are excessively valued [17]. Similarly, in gaming disorder, research has highlighted the overvaluation of game rewards as a critical factor [18]. Additionally, studies on internet addiction have revealed that greater value placed on internet use is positively correlated with problematic internet use [19, 20]. Thus, overvaluation may also be an important mechanism in understanding PSMU.

A recent process-based proposal in behavioral addictions [21], offers a theoretical framework for understanding these disorders, including PSMU. This framework involves two key mechanisms: domain-specific compulsivity and abnormal relative outcome utility computation. According to Perales et al. [21], abnormal outcome utility computation is the main mechanism driving problematic behaviors such as PSMU. Essentially, individuals with this abnormal computation overvalue the rewards linked to a specific behavior. Notably, the authors distinguish between pathological overvaluation and general motivations for engaging in an activity.

In the context of SM, users can engage with these platforms for a variety of reasons, deriving both positive and negative reinforcements, such as making new friends or alleviating negative emotions [22, 23]. Dysfunctional motivations can amplify these reinforcements, leading to excessive reliance on SM for gratification, which may increase the likelihood of developing PSMU [24]. For example, this reliance could arise from using SM to cope with negative emotions or to achieve unmet social needs, as research has indicated [24–26]. Therefore, overvaluation can lead users to prioritize SM over potentially healthier alternatives (e.g., talking face-to-face), turning an adequate use of SM into PSMU, despite it being a planned and goal-directed behavior [21, 27]. This could occur when the perceived benefits of using SM to achieve certain utility domains outweigh alternative behaviors [21, 28]. In fact, overvaluation has been proposed as a key factor contributing to the development of PSMU [24].

Although this process occurs among SM users, a clear definition has yet to be established. Provisionally, we define this vulnerability on the basis of Perales et al. [21] and Redish et al. [29]. Overvaluation of the relative utility of social media (ORUSM) is described as a vulnerability in the decision-making process where the perceived value of using SM to achieve certain utility domains significantly exceeds that of alternative behaviors, leading to excessive selection and heightened reliance on these platforms for gratification.

Several utility domains susceptible to ORUSM, including emotional regulation, social interaction, remaining informed, social identity, social acceptance, and skill development, have been identified [30]. These utility domains have been documented in other studies and form the foundation for the present research [31, 32].

Qualitative studies suggest that some users may overly prefer certain domains, such as communication or emotional regulation, prioritizing SM use for entertainment over other activities such as playing sports or reading [30, 33, 34]. Quantitative research has revealed positive associations between preferences for smartphones over other behaviors and neuroticism, impulsivity, anxiety, depression, and loneliness [34]. Furthermore, there is a positive link between the desire to communicate through SM and PSMU, psychological distress and a negative link with well-being [27, 35, 36]. This phenomenon has been particularly studied in adolescents, as this group represents the most active users of SM [37]. During this developmental stage, people are especially vulnerable to technology-related problematic behaviors, which are strongly linked to various psychological problems [3].

While the reasons behind SM use among adolescents have been extensively explored [22, 38], it is important to distinguish that the utility domains for using SM do not necessarily indicate ORUSM. Users may engage with SM for specific purposes, but this does not automatically imply that they overvalue it. The supporting data highlight this distinction. For example, studies analyzing the relationship between motives for use and adverse psychological outcomes are mainly nonsignificant and weak [31], especially those that focus on learning new skills or looking for information [30]. This contrasts with the stronger associations found in studies focused on ORUSM, as discussed in the previous paragraph.

Given the absence of a psychometrically sound scale in the current literature, this study aims to develop a new instrument, the Plan-net-25 scale, to assess ORUSM in adolescents. Furthermore, the psychometric properties of the newly developed instrument will be thoroughly examined, following utility domains found in Ciudad-Fernández et al. [30]. Specifically, the aim of this study is twofold: (a) to develop a scale specifically designed to measure ORUSM and (b) to assess its psychometric properties.

## Method

### Participants

#### Expert description

In the development of the scale, 14 experts participated in the first round of the Delphi study, and one expert dropped out in the second round. The inclusion criteria for an expert were being fluent in Spanish and performing at least one of the following requisites: (1) having two years of clinical practice in addiction with or without substance, (2) having at least two Journal Citation Reports (JCR)-indexed articles as the first author on behavioral addictions or (3) having methodological studies in psychometric scales. See Table 1 for the characteristics of the experts.

**Table 1:** Information about each participant in the Delphi method

Participants	City	Country	Q1	Q2	Q3
1	Bilbao	Spain	No	No	No
2	Lleida	Spain	Yes	Yes	No
3	Madrid	Spain	Yes	Yes	No
4	Madrid	Spain	No	No	Yes
5	Valencia	Spain	Yes	No	No
6	Valencia	Spain	No	No	Yes
7	Valencia	Spain	Yes	Yes	Yes
8	Louvain	Belgium	Yes	Yes	Yes
9	Melbourne	Australia	No	Yes	Yes
10	Sevilla	Spain	Yes	Yes	No
11	Granada	Spain	No	No	No
12	Madrid	Spain	No	Yes	No
13	Granada	Spain	No	No	No
14	Huelva	Spain	No	No	Yes

Note. Q1 = Two years of clinical practice in behavioral or substance addiction; Q2 = Two years of clinical practice with adolescents; Q3 = Expertise in methodology

#### Pilot study and group interviews

First, 17 adolescents aged 12–17 years from both public and private secondary schools participated in the pilot study. The mean age was 15.88 years, 70.6% self-identified as female, and 29.4% self-identified as male.

Subsequently, cognitive interviews were conducted to assess the generated items. The first group interview was composed of eight participants, and the second consisted of two participants. Seven participants self-identified as female, and three self-identified as male, with ages ranging from 14 to 17 years (*n* = 10).

#### Final sample for assessing the psychometric properties of the Plan-net 25 scale

The sample used to explore the psychometric properties of the Plan-net 25 scale consisted of 2,477 participants: 49% self-identified as male, 49% as female, and the remaining 2% of participants answered different options (i.e., nonbinary, other or prefer not to answer). The average age of the sample was 14.90 years, ranging from 12 to 20 years. Most participants were students from secondary education schools in Valencia, Spain. The inclusion criteria for participants in the study were having access to SM, being fluent in Spanish and being between 12 and 20 years old. Table 2 presents the sociodemographic characteristics of the participants.

**Table 2:** Descriptives of the sample used in the validation of the Plan-net 25 scale

Variable	*N* = 2,477^1^
**Age**	14.90 (2.49)
**Grade**	
1st year of Compulsory Secondary Education	506 (18%)
2nd year of Compulsory Secondary Education	477 (17%)
3rd year of Compulsory Secondary Education	501 (18%)
4th year of Compulsory Secondary Education	465 (17%)
1st year of Baccalaureate	255 (9.3%)
2nd year of Baccalaureate	218 (7.9%)
Intermediate Vocational Training	202 (7.3%)
Advanced Vocational Training	95 (3.5%)
Other	33 (1.2%)
**City**	
Valencia	2,487 (90%)
Madrid	273 (9.9%)
**Gender Identity**	
Boy	1,341 (49%)
Girl	1,354 (49%)
Nonbinary	13 (0.5%)
Other	4 (0.1%)
Prefer not to answer	38 (1.4%)
**Nationality**	
Spanish	2,136 (86%)
Other	336 (14%)
**Do you have access to social media on your phone**,** computer**,** or your parents’/guardians’ device?**	
Yes	2,760 (100%)
No	0 (0%)
**Do you frequently use social media**,** such as WhatsApp**,** TikTok**,** Instagram**,** Twitch**,** YouTube, etc., more than 3 times a week?**	
Yes	2,669 (97%)
No	84 (3.1%)

Note. Mean (SD); n (%)

### Measures

Table 3 shows the questions and scales administered, the original validation, the Spanish adaptation used, and the factors comprising each scale. Cronbach’s α and McDonald’s ω are included as indicators of internal consistency (ranging from 0.87 to 0.94).

**Table 3:** *Description of the study measures*

Domain assessed	Instrument	Variable	Description	Reliability
**Demographics**				
Gender	Ad hoc scale	Gender	Boy, girl, nonbinary, other, or prefer not to answer.	NA
Age	Ad hoc scale	Age	Age	NA
Grade	Ad hoc scale	Grade	1st, 2nd, 3rd, or 4th year of ESO (Compulsory Secondary Education), 1st or 2nd year of Baccalaureate (High School), Intermediate Vocational Training, Higher Vocational Training, other (For example, Basic Vocational Training).	NA
City	Ad hoc scale	City	Valencia, Madrid, or another location.	NA
Nationality	Ad hoc scale	Nationality	Spanish or other.	NA
SM access	Ad hoc scale	SM access	Do you have access to social media on your phone, computer, or your parents’/legal guardians’ device? When we refer to social media, we mean platforms such as Instagram, WhatsApp, TikTok, Twitter, or BeReal.	NA
Frequent use of SM	Ad hoc scale	Social Media Frequency of use	Do you frequently use social media such as WhatsApp, TikTok, Instagram, Twitch, YouTube, etc., more than 3 times a week? You can use them on your parents’/legal guardians’ phone or your friends’ phone, even if you do not have access on your own phone.	NA
**Problematic SM use**				
ORUSM	Plan-net 25 scale	Plan-net 1	This scale aims to measure adolescents’ overvaluation of the use of social media through a scale rated on a 6-point Likert scale (0 = Completely disagree, 5 = Completely agree). The psychometric properties of this scale will be studied in the present study. It is composed of 7 different dimensions. This first dimension assesses the overvaluation of the relative utility of SM for social interaction (e.g., If I did not have access to social media, I would have a lot of difficulty communicating with people my age).	α = 0.87, ω = 0.89
		Plan-net 2	Overvaluation of the relative utility of SM for meeting new people (e.g., if I did not have access to social media, I would have a lot of difficulty making new friends).	α = 0.88, ω = 0.88
		Plan-net 3	Overvaluation of the relative utility of SM for regulating unpleasant emotions (e.g., if I did not have access to social media, I would have a lot of difficulty reducing my stress).	α = 0.93, ω = 0.94
		Plan-net 4	Overvaluation of the relative utility of SM for feeling socially accepted (e.g., if I did not have access to social media, I would have a lot of difficulty feeling included in my group of friends).	α = 0.93, ω = 0.93
		Plan-net 5	Overvaluation of the relative utility of SM for keeping up with what is happening (e.g., if I did not have access to social media, I would have a lot of difficulty finding out what people in my surroundings are doing).	α = 0.88, ω = 0.93
		Plan-net 6	Overvaluation of the relative utility of SM for expressing oneself socially (e.g., if I did not have access to social media, I would have a lot of difficulty expressing my feelings).	α = 0.89, ω = 0.91
		Plan-net 7	Overvaluation of the relative utility of SM for managing boredom (e.g., if I did not have access to social media, I would have a lot of difficulty keeping myself entertained.).	α = 0.91, ω = 0.92
Problematic Use of Social Media	Social Media Disorder Scale (SMD [39, 40])	Problematic Use of Social Media	The SMD scale is composed of 9 items that evaluate problematic use of social media (e.g., have you regularly felt dissatisfied because you wanted to spend more time on social media?). Although the original validation scale used a dichotomous response scale (Yes/No), following the example of the validation in the Turkish population by Savci et al. [41], we adapted the response scale to a Likert scale (1–6). We employed a 6-point Likert scale (1 = Completely disagree, 6 = Completely agree). Higher scores indicate greater problematic use.	α = 0.89, ω = 0.91
**Psychological variables**				
Depression	Patient Health Questionnaire (PHQ-9 [42, 43])	Depression symptoms	The PHQ questionnaire is composed of 9 items measuring depression (e.g., little interest or pleasure in doing things). Each item is scored from 0 (not at all) to 3 (nearly every day), with higher scores indicating greater severity. This version was extracted from the Patient Health Questionnaire Screeners website and adapted from the Spanish version by Diez-Quevedo et al. [44] for use with the adolescent population.	α = 0.91, ω = 0.93
Anxiety	Generalized Anxiety Disorder (GAD-7 [45, 46])	Generalized anxiety symptoms	The GAD consists of 7 items assessing generalized anxiety disorder symptoms (e.g., feeling nervous, anxious, or on edge). Items are rated on a 4-point Likert scale (0 = never; 3 = almost every day). Higher scores indicate greater severity of symptoms.	α = 0.92, ω = 0.94
Satisfaction With Life Scale	Satisfaction With Life Scale (SWLS-3; [47, 48])	Satisfaction with life	This scale is composed of 3 items and measures overall life satisfaction (e.g., the conditions of my life are excellent). Items are scored on a 7-point Likert scale (1 = strongly disagree; 7 = strongly agree). Higher scores on this scale indicate greater satisfaction with life. Originally, this scale consisted of 5 items. However, we followed Kjell & Diener’s [49] recommendations and removed the last two items.	α = 0.87, ω = 0.87
Self-esteem	Single-Item Self-Esteem Scale (SISE; [50, 51])	Self-esteem	This measure assesses self-esteem with one item (i.e., I have high self-esteem). This item employs a 6-point Likert scale ranging from 0 to 5 (0 = none of the time; 5 = all the time). Higher scores indicate higher self-esteem. Because the scale consists of one item, internal consistency cannot be computed.	NA
Loneliness	Three-Item Loneliness Scale (TILS [52, 53])	Loneliness	This questionnaire assesses perceived loneliness using 3 items (e.g., how often do you feel left out?). This scale employs a 3-point Likert scale ranging from 1 (hardly ever) to 3 (often), with higher scores indicating greater loneliness.	α = 0.89, ω = 0.91

Note: ^a^ = Spanish translation and validation; NA = Non-Applicable

### Procedure

First, to identify a representative sample of Spanish-speaking experts for the Delphi study, a comprehensive review of recently published papers in high-impact journals (e.g., Journal of Behavioral Addictions or Addictive Behaviors) was conducted to identify publications by Spanish-speaking authors. Experts in addiction methodology or clinical practice were contacted and selected on the basis of prior collaborations, influence, or connections with experts. Methodologists focused on addiction research. Emphasis was placed on ensuring representation from different regions and countries to achieve broad geographical and cultural diversity. However, challenges were encountered in recruiting Spanish-speaking experts from South America, as only one South American expert was recruited (working in Belgium).

A pilot study was then conducted. Seventeen 12- to 17-year-old participants provided online feedback on the scale and assessed the comprehensibility of the items. Additionally, two groups of cognitive interviews with adolescents were held to further examine validity on the basis of response processes. Cognitive interviews were chosen as a qualitative method to examine how respondents understood, interpreted, and responded to the scale. The first cognitive interview included eight participants, and the second included two participants.

Once the final version of the scale had been developed, education centers were contacted to take part in the study. Participation was not compensated, but it was incentivized by means of a personalized report of the results obtained from the survey. Data collection was conducted in person, either on paper or digitally (e.g., tablet, smartphone, computer). The responses collected digitally were entered directly into the Qualtrics platform, whereas the paper responses were later input into Qualtrics by researchers. The data collection dates ranged from September 2023 to May 2024. The procedures for scale development and sample recruitment are detailed in the supplementary material.

### Statistical analysis

#### Delphi study evaluation of the Plan-net-25 scale

The experts evaluated each item in each round via a Likert scale ranging from 1 (minimal) to 4 (extremely) to assess its suitability. The characteristics of the items were clarity, appropriateness, and relevance. To decide whether to retain or remove an item, the content validity index (I-CVI) was calculated for each item within each subdimension of the Plan-net 25, and to evaluate the scale, the content validity index for scale (S-CVI) was calculated [54]. The I-CVI was determined by the proportion of experts who rated the item as 3 or 4. The S-CVI was calculated by averaging the values of all the I-CVI. The recommended and employed cutoff point in this study for groups of this size was 0.78 [55], although different values have been proposed in the literature (e.g., 0.80 [56]).

#### Prior calculation of the sample size

Before recruiting, the sample size was determined to be at least 1,750 participants via pwrSEM [57]. Details are provided in the supplementary materials.

#### Data cleaning and internal consistency

Once the scales were administered to the target sample, data cleaning was conducted. Participants who answered incorrectly more than one out of three control questions (e.g., ‘If you are paying attention, mark ‘Somewhat Disagree’; *n* = 250), following Buchanan & Scofield’s [58] proposal, were removed. Those who did not have access to SM were also excluded (*n* = 82), leaving a total sample of 2,477. Frequencies and descriptive statistics were computed. Afterwards, the total sample was randomly divided into two segments: 800 for exploring the latent structure and the rest for evaluating internal consistency and associations and confirming the latent structure, ensuring that the sample size complied with the power analysis criteria for confirmatory factor analysis set forth in the simulation. Internal consistency was subsequently assessed via Cronbach’s α and McDonald’s ω via polychoric correlation matrices for items with four or fewer response categories.

#### Latent structure of the Plan-net 25 scale

Exploratory graph analysis (EGA) using the ‘Louvain’ and ‘Walktrap’ algorithms, along with parallel analysis, was conducted to explore latent structures of the Plan-net 25 scale via different methods.

Several confirmatory factor analyses (CFA) were subsequently conducted to evaluate different latent structures of the Plan-net 25 scale. First, a one-factor model was tested to evaluate whether ORUSM is a unidimensional construct, as proposed by Redish [9]. On the basis of the results of the EGA using the Walktrap algorithm, a six-factor model with correlated factors (clustering both the 1st and 2nd original utility domains) was tested. Next, a seven-factor model with correlated factors was evaluated, as suggested by parallel analysis, the EGA Louvain algorithm, and the final decision in the second round of the Delphi study. Finally, owing to the high correlation values between factors in the seven-factor model, a hierarchical seven-factor model was tested. The model fit was evaluated via several criteria: the χ² statistic and its associated *p* value, the comparative fit index (CFI), and the Tucker‒Lewis index (TLI), with values above 0.95 considered excellent and those above 0.90 acceptable. Additionally, the Root Mean Square Error of Approximation (RMSEA) and its 90% confidence intervals were considered adequate if they were less than 0.06, and the Standardized Root Mean Square residual (SRMR) was considered acceptable with a cutoff close to 0.08, following the guidelines set by Hu and Bentler [59]. The Akaike Information Criterion (AIC), Bayesian Information Criterion (BIC) and Sample-Size Adjusted Bayesian Information Criterion (SABIC) were also reported, with lower values indicating a better fit. The χ² statistic was reported but not interpreted due to its high sensitivity to sample size [60]. Models were estimated via Maximum Likelihood Estimation with robust standard errors (MLR), as multivariate normality was not met.

#### Measurement and structural invariance testing

The measurement model invariance of the scale was tested across age groups (12–15 and 16–20 years) and between male and female participants. Owing to the limited number of respondents selecting other gender options, these respondents were excluded from the gender invariance analyses. Invariance testing for Plan-net 25 was conducted via the 7-factor model, following a hierarchical approach: (1) testing the 7-factor model for each group, (2) configural model without restrictions, (3) constraining item factor loadings, (4) item intercepts, (5) residual errors, and (6) latent factor means. The relevant misfit was determined by *p* values for χ² changes, as well as changes in CFI and RMSEA. A change of more than 0.03 in RMSEA and 0.02 in CFI was considered significant when testing constrained item factor loadings, with stricter thresholds of 0.01 for further constraints, on the basis of Rutkowski & Svetina [61]. However, changes in CFI and RMSEA were prioritized over χ², given the latter’s sensitivity to reject true models with large sample sizes [60].

To compare scores across invariance groups, factor scores were used, and the regression method was applied for each Plan-net 25 factor to ensure a more precise comparison among groups [62]. Differences between boys and girls, as well as between early and late adolescents, were then assessed via t-tests. Bonferroni correction was applied to minimize Type I errors. Results are available in the supplementary material.

#### Correlation analysis

Correlations were computed to explore the convergent validity of the Plan-net 25 scale. Previously, CFA were conducted on each scale (see supplementary material). Then, factor scores were obtained via the regression method for each subscale. This approach was adopted to derive a more accurate correlation matrix to construct a network psychometric model [62]. Thus, associations between the Plan-net-25 subscales and other relevant variables within the context of PSMU, such as life satisfaction, depression, loneliness, anxiety, and self-esteem, were examined through polychoric correlations. Since self-esteem was evaluated via a single-item measure, factor scores were not available, and raw scores were employed.

#### Network analysis

Later, a psychometric network analysis was conducted, where items (nodes) are connected by edges representing partial correlations between them, adjusted for all other nodes [63]. The Fruchterman-Reingold algorithm was used to position nodes, with stronger connections placed closer together [64].

First, a polychoric correlation matrix was computed using factor scores for all scales and the raw score for the item assessing self-esteem. Then, graphical Least Absolute Shrinkage and Selection Operator (gLASSO) regularization was applied, following the Extended Bayesian Information Criterion (EBIC) with a default hyperparameter of 0.05. In this network visualization, positive and negative correlations are depicted by green and red edges, respectively, with edge thickness indicating the strength of the associations [65]. Furthermore, the proportion of variance explained by the model at each node was determined via the R² statistic, which was visually represented by a pie chart within each node.

Finally, the robustness of the network was assessed via a nonparametric bootstrap approach with 5,000 iterations to determine network stability and a case-dropping subset bootstrap, with 5,000 iterations, to gauge the stability of centrality indices. The stability coefficient was computed for this purpose. This coefficient reflects the proportion of the sample that can be excluded while still ensuring that the centrality indices’ correlation with those derived from the bootstrapped samples remains at or above 0.70 within a 95% confidence interval. A stability coefficient above 0.25 was considered acceptable, and above 0.50, it was considered satisfactory [66]. Power analysis conducted on the network indicated adequate sensitivity and specificity.

All analyses were conducted in R 4.3.2, and the following packages were employed to perform the analyses: *Psych*, *gtsummary*, *lavaan*, *semTools*, *bootnet*, *qgraph*, *mgm*, *EGAnet* and *rstatix* [67–74].

### Ethics

The Ethics Committee of the University of Valencia approved the study (Procedure number 2039883). This study was conducted in accordance with the Declaration of Helsinki. Before recruitment began, participants received information about the study’s objectives and provided their informed consent. For participants aged 14 and older, consent included the option to provide their birth date and initials to facilitate follow-up. Parents or legal guardians were informed of the study through each participating school, following a passive informed consent procedure. For participants under 14, data collection was completely anonymous. This study was preregistered in April 2022 on the Open Science Framework (https://osf.io/wc4ev/?view_only=721ad5a81af944d886d82a1ab742f805). Data, scripts, and supplementary and other materials are available on the same OSF page.

## Results

### Scale development

Initially, a set of initial items was generated to assess ORUSM. To judge the quality of the generated items, the Delphi methodology was employed. In the first round, the experts were presented with a pool of 55 items, which were refined by the lead researcher. All the items shared the common header “Social media are the best way to… and varied according to the specific utility domain they addressed (e.g., social interaction, emotional regulation, social approval, staying informed, identity and social expression, information seeking, and skill development). Afterward, the experts’ suggestions were incorporated, and they received anonymous feedback on their general ratings for each item.

In the second round, 54 items were presented to the experts, most of which were either new or reformulated. A new common header was introduced: “If I didn’t have social media, I would have a lot of difficulty…” This change followed an expert’s suggestion to reduce false positives and evoke a craving state (hot state) when responding. ORUSM can be divided into seven specific utility domains: (1) interacting socially, (2) meeting new people, (3) regulating unpleasant emotions, (4) feeling socially accepted, (5) keeping up with what is happening, (6) expressing oneself socially, and (7) managing boredom. Managing boredom was included to distinguish it from emotional regulation. Additionally, social interaction was split into two related but distinct domains: interacting socially and meeting new people. Finally, skill development was removed because of its limited clinical utility.

As a result, 27 items with the best I-CVI values were selected. Table 4 shows the I-CVI values for clarity, appropriateness, and relevance in the various phases of the scale’s development with the expert group. The experts also conducted a qualitative assessment of the instrument’s instructions and the chosen Likert scale format, showing general agreement with the 6-point Likert scale.

**Table 4:** S-CVI regarding clarity, appropriateness and relevance

	Clarity	Appropriateness	Relevance
1st round (54 items)	0.89	0.85	0.81
2nd round (55 items)	0.91	0.92	0.90
2nd round after removing items (27 items)	0.95	0.98	0.96

A pilot study with adolescents was subsequently conducted, followed by two cognitive interviews to assess validity on the basis of response processes. This approach improved the comprehensibility of the items. To create the final version of the instrument, a minimum of at least 3 items per utility domain was needed. All this information is detailed in the supplementary material.

### Descriptive statistics

Table 5 shows the average scores and standard deviations of the participants on each of the scales used. No significant differences were found between the randomly selected sample of 800 participants and the remaining participants. Plan-net 5 and Plan-net 7 show higher mean values compared to the remaining subscales of Plan-net 25. This suggests that adolescents on this sample have greater difficulty staying informed and managing boredom if they did not have access to SM. The English and Spanish versions of the Plan-net 25 scale are provided in the supplementary materials.

**Table 5:** Descriptive statistics for the general sample

Variables	*N* = 2,477^1^
Plan-net1	1.92 (1.29)
Plan-net2	1.60 (1.21)
Plan-net3	1.62 (1.28)
Plan-net4	1.43 (1.27)
Plan-net5	2.82 (1.19)
Plan-net6	1.41 (1.11)
Plan-net7	2.30 (1.37)
SMD	19.62 (10.05)
PHQ	7.34 (6.07)
GAD	5.8 (5.2)
SWLS	5.09 (1.40)
TILS	4.15 (2.16)
SE	3.42 (1.19)

Note. ^1^ = Mean (SD). Plan-net1: ORUSM for interacting socially; Plan-net2: ORUSM for meeting new people; Plan-net3: ORUSM for regulating unpleasant emotions; Plan-net4: ORUSM for feeling socially accepted; Plan-net5: ORUSM for keeping up with what is happening; Plan-net6: ORUSM for expressing oneself socially; Plan-net7: ORUSM for managing boredom; SMD: problematic social media use; PHQ: depression; GAD: anxiety; SWLS: life satisfaction; TILS: loneliness; SE: self-esteem

### Factor analysis and measurement invariance

Table 6 presents various factor models, along with measurement invariance between boys and girls and between those under 16 years old (early adolescents) and those 16 years old or older (late adolescents). The one-factor model showed a poor fit to the data (χ²(275) = 10389.001, *p* <.001, CFI = 0.554, TLI = 0.514, RMSEA = 0.165 [0.162, 0.168], SRMR = 0.103). Similarly, the six-factor model also provided a poor fit (χ²(260) = 2701.073, *p* <.001, CFI = 0.898, TLI = 0.882, RMSEA = 0.081 [0.079, 0.084], SRMR = 0.066). In contrast, the theoretical seven-factor model demonstrated the best fit for the data (χ²(254) = 1633.405, *p* <.001, CFI = 0.942, TLI = 0.931, RMSEA = 0.062 [0.059, 0.065], SRMR = 0.057). Lastly, the seven-factor model with a second order factor also provided an adequate fit (χ²(268) = 1941.987, *p* <.001, CFI = 0.930, TLI = 0.921, RMSEA = 0.066 [0.064, 0.069], SRMR = 0.067). The best fitting model was retained for subsequent analysis (i.e., seven-factor model).

**Table 6:** *Factor models for the Plan-net-25 scale*,* including invariance models for age groups and boys and girls*

Model	χ²	df	*p* value	CFI	TLI	RMSEA	SRMR	AIC	BIC	SABIC	Δχ²	Δ df	*p* value comparison	Δ CFI	Δ RMSEA
Unifactorial Model	10389.001	275	< 0.001	0.554	0.514	0.165 [0.162, 0.168]	0.103	147723.328	148138.315	147900.041	-	-	-	-	-
6-Factor Model	2701.073	260	< 0.001	0.898	0.882	0.081 [0.079, 0.084]	0.066	136989.418	137487.402	137201.474	-	-	-	-	-
7-Factor Model	1633.405	254	< 0.001	0.942	0.931	0.062 [0.059, 0.065]	0.057	135605.942	136137.125	135832.135	-	-	-	-	-
Hierarchical 7-Factor Model	1941.987	268	< 0.001	0.930	0.921	0.066 [0.064, 0.069]	0.067	135982.186	136435.905	136175.392	-	-	-	-	-
Boys Only	908.476	254	< 0.001	0.942	0.931	0.061 [0.057, 0.065]	0.052	66070.348	66532.333	66227.450	-	-	-	-	-
Girls Only	1041.650	254	< 0.001	0.939	0.927	0.065 [0.061, 0.069]	0.064	69455.076	69922.301	69617.408	-	-	-	-	-
Gender Invariance: Configural	1949.886	508	< 0.001	0.940	0.929	0.063 [0.060, 0.066]	0.058	135525.423	136587.790	135977.809	-	-	-	-	-
Gender Invariance: Loadings	1974.837	526	< 0.001	0.940	0.932	0.062 [0.059, 0.065]	0.059	135507.065	136469.835	135917.039	16.360	18	0.567	0.000	0.001
Gender Invariance: Intercepts	2016.673	544	< 0.001	0.940	0.933	0.061 [0.058, 0.064]	0.059	135506.321	136369.494	135873.885	34.945	18	0.009	0.001	0.000
Gender Invariance: Residuals	2057.315	569	< 0.001	0.938	0.934	0.061 [0.058, 0.063]	0.059	135561.499	136286.343	135870.158	56.320	25	< 0.001	0.001	0.001
Gender Invariance: Latent Means	2098.346	576	< 0.001	0.937	0.934	0.061 [0.058, 0.064]	0.062	135593.396	136279.508	135885.562	45.662	7	< 0.001	0.001	0.000
Early Adolescents	1038.278	254	< 0.001	0.947	0.938	0.058 [0.055, 0.062]	0.057	88093.096	88582.699	88277.764	-	-	-	-	-
Late Adolescents	845.988	254	< 0.001	0.935	0.924	0.066 [0.061, 0.071]	0.058	47464.229	47895.047	47590.246	-	-	-	-	-
Age Invariance: Configural	1891.024	508	< 0.001	0.943	0.933	0.061 [0.058, 0.064]	0.057	135557.326	136619.692	136009.711	-	-	-	-	-
Age Invariance: Loadings	1922.709	526	< 0.001	0.943	0.935	0.060 [0.057, 0.063]	0.057	135546.750	136509.519	135956.724	24.136	18	0.151	0.001	0.000
Age Invariance: Intercepts	1991.679	544	< 0.001	0.941	0.935	0.060 [0.057, 0.063]	0.058	135580.264	136443.437	135947.828	69.844	18	< 0.001	0.002	0.000
Age Invariance: Residuals	2009.001	569	< 0.001	0.940	0.937	0.059 [0.057, 0.062]	0.058	135603.854	136328.698	135912.513	39.697	25	0.031	0.000	0.001
Age Invariance: Latent Means	2046.623	569	< 0.001	0.939	0.937	0.060 [0.057, 0.062]	0.061	135630.649	136316.761	135922.815	41.778	7	< 0.001	0.001	0.000

Note. The scaled χ² statistic and the robust versions of the comparative fit index (CFI), Tucker‒Lewis index (TLI), and root mean square error of approximation (RMSEA) statistics have been reported. However, for the comparison of nested models, a comparison was made between the two standard chi-square values. This is because a robust difference test is a function of two standard (nonrobust) statistics, as indicated by the package used to perform invariance testing in the CFA models (SemTools)

Table 7 shows the observed factor loadings. All factor loadings exceed the traditional cutoff point (i.e., 0.40). However, items 1 and 4 from Plan-net 5 and item 1 from Plan-net 6 obtained factor loadings lower than 0.70.

Furthermore, the correlations between factors range from 0.39 (between factor 2 and factor 3) to 0.65 (between factor 2 and factor 6). All the factor correlations are positive and significant. The supplementary materials provide the values of the associations between the factors in the 7-factor model.

**Table 7:** Factor loadings of each item

Item number	Items	Standardized factor loading
	**ORUSM for interacting socially**	
1	Communicating with people my age	0.754
2	Chatting with my friends.	0.822
3	Staying in touch with my classmates	0.714
4	Meeting up with my friends	0.716
	**ORUSM for meeting new people**	
5	Making new friends	0.837
6	Finding people with similar interests (e.g., hobbies, music, sports, etc.).	0.738
7	Meeting new people (e.g., partners, friends, etc.).	0.836
	**ORUSM for regulating unpleasant emotions**	
8	Reducing my stress levels.	0.781
9	Feeling better when I am sad.	0.799
10	Calming myself down when I feel nervous.	0.903
11	Easing my mind when I am overwhelmed.	0.914
	**ORUSM for feeling socially accepted**	
12	Feeling included in my group of friends.	0.931
13	Feeling like a part of my group of friends.	0.927
14	Feeling connected to my social circles (e.g., friends, family, etc.).	0.765
	**ORUSM for keeping up with what is happening**	
15	Staying updated with current news and events.	0.681
16	Finding out what people around me are doing.	0.874
17	Learning about what others (e.g., friends, colleagues, family members, etc.) are doing.	0.843
18	Keeping up with what is going on in the world.	0.685
	**ORUSM for expressing oneself socially**	
19	Sharing my interests with others (e.g., friends, colleagues, family members, etc.).	0.654
20	Expressing my emotions.	0.849
21	Expressing my thoughts.	0.879
22	Giving my opinion on a topic.	0.733
	**ORUSM for managing boredom**	
23	Keeping myself entertained.	0.854
24	Hanging out.	0.910
25	Having fun.	0.813

Note. Plan-net1: ORUSM for interacting socially; Plan-net2: ORUSM for meeting new people; Plan-net3: ORUSM for regulating unpleasant emotions; Plan-net4: ORUSM for feeling socially accepted; Plan-net5: ORUSM for keeping up with what is happening; Plan-net6: ORUSM for expressing oneself socially; Plan-net7: ORUSM for managing boredom. The common header for the items is “If I didn’t have access to social media, I would have a lot of difficulty…”. However, we recommend using only “If I did not have access to social media” because in cognitive interviews, participants start answering similarly to a motives scale

Invariance testing between boys and girls was conducted step-by-step, adding constraints progressively to assess different levels of invariance. In the initial model, no constraints were applied, yielding a good fit to the data. The next model constrained factor loadings to be equal across genders, and this model maintained an acceptable fit (*p* =.567 and with no relevant changes in ΔCFI and ΔRMSEA). When intercepts were further constrained, the χ² difference test indicated a significant increase in misfit (*p* =.009), but changes in ΔCFI and ΔRMSEA remained below the 0.01 threshold. Constraining residual variances also led to a significant χ² change (*p* <.001), though the ΔCFI and ΔRMSEA changes were minimal. Finally, constraining latent means resulted in another significant χ² increase (*p* <.001), but with ΔCFI and ΔRMSEA values still within acceptable limits.

The same approach was applied to test invariance between early and late adolescents. Initially, no constraints were imposed and the model provided a good fit to the data. Constraining factor loadings in the second model yielded an acceptable fit (*p* =.151 and with no relevant changes in ΔCFI and ΔRMSEA). When intercepts were constrained, a significant misfit emerged in the χ² difference test (*p* <.001), but ΔCFI and ΔRMSEA remained under the 0.01 threshold. Constraining residual variances in the subsequent model again produced a significant χ² increase (*p* =.032), but with minimal changes in ΔCFI and ΔRMSEA. Finally, constraining latent means resulted in another significant χ² difference (*p* <.001), but fit indices remained within acceptable bounds.

In the invariance tests for both gender and age groups, the factor structure remained consistent across groups. Constraining factor loadings, intercepts, residuals, and latent means resulted in only minimal changes in CFI and RMSEA, despite significant χ² difference tests. This indicates that the measurement properties of the model are comparable across groups, ensuring that observed differences are due to actual differences in the construct rather than measurement bias.

### Correlation analyses

**Fig. 1:**
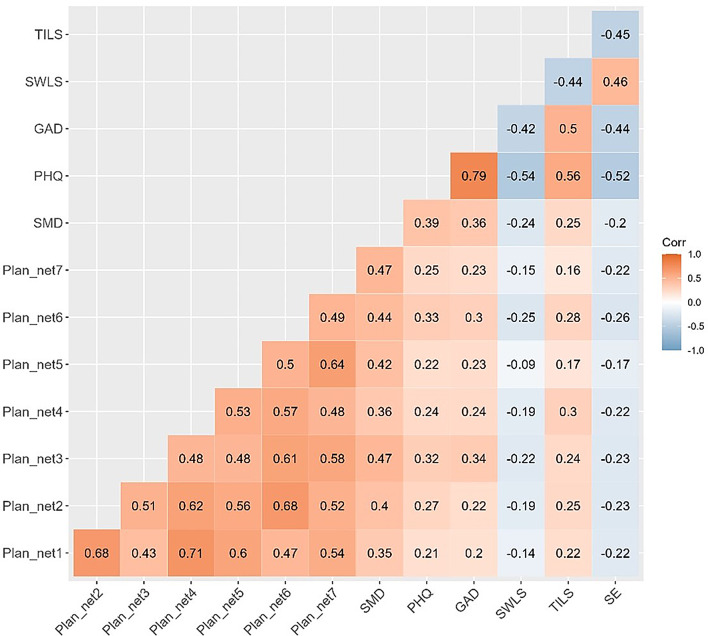
Polychoric correlation matrix using factor scores. Note. Plannet1: ORUSM for interacting socially; Plannet2: ORUSM for meeting new people; Plannet3: ORUSM for regulating unpleasant emotions; Plannet4: ORUSM for feeling socially accepted; Plannet5: ORUSM for keeping up with what is happening; Plannet6: ORUSM for expressing oneself socially; Plannet7: ORUSM for managing boredom; SMD: problematic social media use; PHQ: depression; GAD: anxiety; SWLS: life satisfaction; TILS: loneliness; SE: self-esteem

Figure 1 presents a matrix of polychoric correlations, showing a positive association between depression, anxiety, loneliness, and PSMU with all Plan-net 25 subdimensions (ranging from 0.16 to 0.47). In contrast, negative associations are observed between these subscales and self-esteem and life satisfaction (ranging from − 0.09 to − 0.26). Overall, the correlations of PSMU with depression, anxiety, loneliness, life satisfaction, and self-esteem are slightly stronger than those between the Plan-net 25 scales and these psychological constructs.

### Network analysis

The sample size is 1798 participants for this network. The network consists of 13 nodes, with a density of 31 out of 78 possible connections. There is a clear separation between two components: on one side are the psychological variables (i.e., depression, anxiety, loneliness, self-esteem, and life satisfaction), and on the other side are the subscales of the Plan-net 25 scale and PSMU, which are highly connected. This analysis reveals strong associations within the Plan-net 25 subscales. Plan-net 1 (ORUSM for interacting socially) shows strong positive associations with Plan-net 2 (ORUSM for meeting new people) and Plan-net 4 (ORUSM for feeling socially accepted). Additionally, Plan-net 2 (ORUSM for meeting new people) is strongly associated with Plan-net 6 (ORUSM for expressing oneself socially). Remarkably, there is a negative association between Plan-net 1 (ORUSM for interacting socially) and Plan-net 6 (ORUSM for expressing oneself socially). Plan-net 5 (ORUSM for finding out what is happening) also shows a strong association with Plan-net 1 (ORUSM for interacting socially), and there is a notable connection between Plan-net 5 and Plan-net 7 (ORUSM for managing boredom). Additionally, Plan-net 3 (ORUSM for regulating unpleasant emotions) displays associations with both Plan-net 6 (ORUSM for expressing oneself socially) and Plan-net 7 (ORUSM for managing boredom).

Network analysis also reveals associations among the Plan-net subscales and indicators of distress, well-being and PSMU. Positive associations are observed between Plan-net 4 (ORUSM for feeling socially accepted) and loneliness, as well as between PSMU and Plan-net 3 (ORUSM for regulating unpleasant emotions), Plan-net 5 (ORUSM for determining what is happening) and Plan-net 7 (ORUSM for managing boredom). Additionally, a positive link between Plan-net 5 (ORUSM for keeping up with what is happening) and life satisfaction is noted. Importantly, no Plan-net nodes are associated with depression. However, Plan-Net 3 (ORUSM for regulating unpleasant emotions) is positively related to anxiety, whereas PSMU is not associated with anxiety.

The psychometric network showed strong stability and robustness. Bootstrap analyses indicated stable connection weights and centrality indices, especially for strength and expected influence. Sensitivity and specificity analysis confirmed that the sample size was sufficient. For further details, see the supplementary material. 

**Fig. 2:**
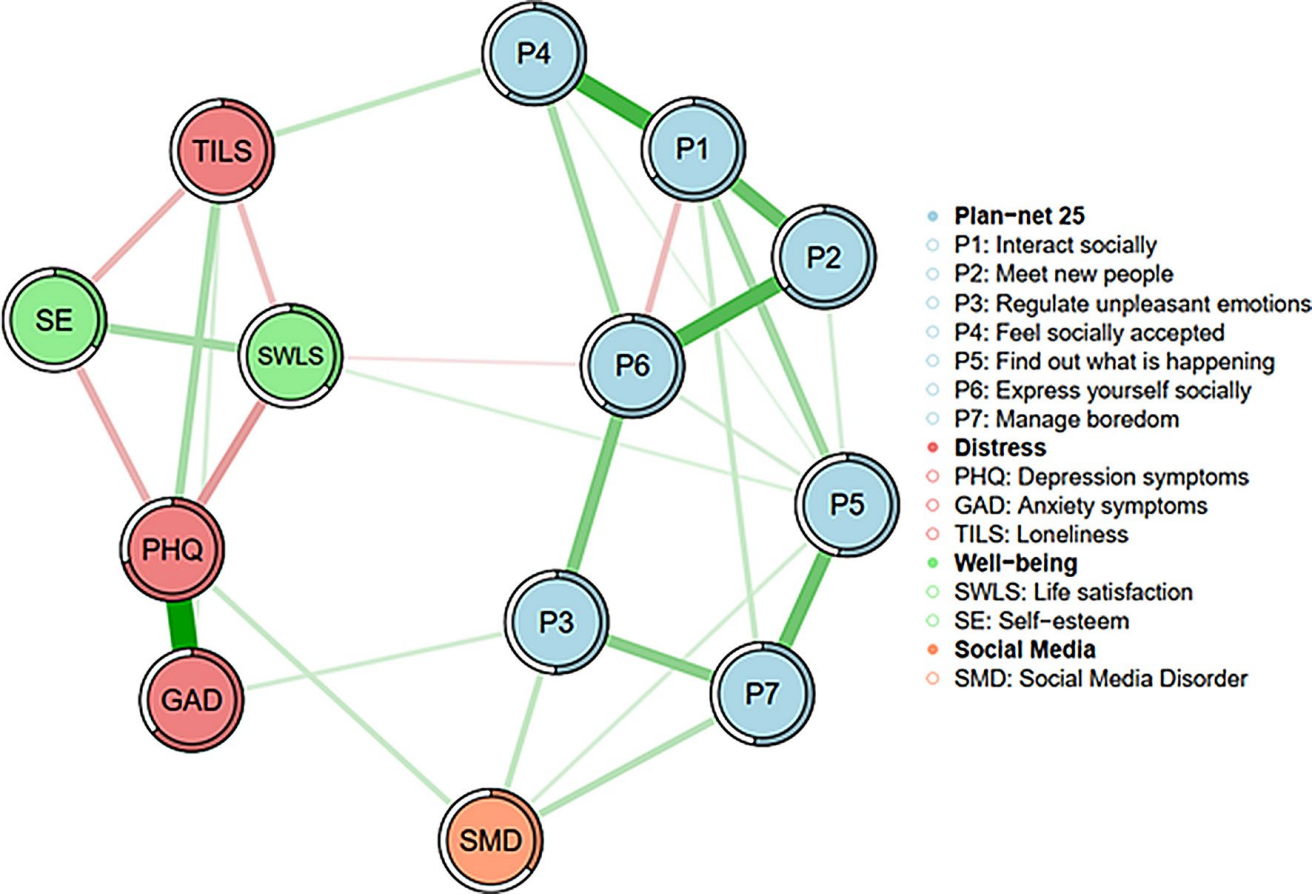
Network analysis of psychological variables. Note. Plannet1: ORUSM for interacting socially; Plannet2: ORUSM for meeting new people; Plannet3: ORUSM for regulating unpleasant emotions; Plannet4: ORUSM for feeling socially accepted; Plannet5: ORUSM for keeping up with what is happening; Plannet6: ORUSM for expressing oneself socially; Plannet7: ORUSM for managing boredom; SMD: problematic social media use; PHQ: depression; GAD: anxiety; SWLS: life satisfaction; TILS: loneliness; SE: self-esteem

## Discussion

The aim of this study was twofold: (a) to develop a scale specifically designed to measure ORUSM and (b) to assess its psychometric properties. A new psychometric instrument, the Plan-net 25 scale, was developed in this study. Additionally, various aspects of reliability and validity were examined in a substantial sample of Spanish adolescents.

The adoption of the Delphi methodology was instrumental in refining the scale items, specifically by modifying the statements to...“If I had not social media, I would have a lot of difficulties to.“, thus addressing potential issues of false positives—a significant concern in PSMU research [75, 76]. This change also allows one to evoke a craving state when one responds to items [77]. Suggestions are also crucial in differentiating between the utility of SM in regulating unpleasant emotions and avoiding boredom, as indicated by previous research [78].

Confirmatory factor analysis did not support either the one-factor model or the six-factor model. The results revealed a 7-factor structure that details various utility domains of ORUSM, resonating with the multifaceted motives for the use of SM. These results align with prior research and the decisions made in the Delphi study [30]. Additionally, the hierarchical 7-factor model demonstrated an acceptable fit, although it was weaker than the 7-factor model. Therefore, the hierarchical latent structure should not be disregarded, as it is supported by previous literature on overvaluation [9, 13, 29]. A final decision on which latent structure is more stable and replicable is still pending.

High internal consistency values across the subscales indicate a low measurement error in each utility domain, enabling focused application in specific contexts (e.g., assessing the fluctuation of ORUSM for entertainment in an experimental setting).

Positive associations between PSMU and all ORUSM subscales provided relevant insights into the potential factors underlying PSMU. These results suggest an increased likelihood of this mechanism being present in individuals exhibiting PSMU, as predicted by theoretical frameworks [9, 13, 21]. These moderate and significant correlations with all these subscales align with the literature that links heightened valuation of addictive behaviors or substances despite the adverse consequences related to engaging in those behaviors [79–81].

Notably, ORUSM for the regulation of unpleasant emotions and seeking entertainment are closely tied to PSMU in our sample. This finding is in line with previous findings that showed that a propensity toward boredom and difficulty in emotional regulation correlated significantly with PSMU [82, 83]. Individuals who excessively use SM to manage emotions or boredom may lack coping flexibility, which hinders their ability to achieve healthy psychological adjustment [84]. Furthermore, negative associations were found between the Plan-net-25 subscales and well-being indicators, and positive associations were found with distress indicators, paralleling the relationships between PSMU and these factors. This pattern has been replicated in studies examining the preference for SM, which is correlated with impulsivity, PSMU, neuroticism, anxiety, depression, and loneliness [85, 35]. Moreover, the preference for online communication over face-to-face interaction has been positively associated with loneliness and emotional problems and negatively linked to life satisfaction and physical health [27, 36, 86]. These findings have been replicated in gaming disorder, where overvaluation of gaming rewards is modestly linked to depression and anxiety [87], suggesting that it is just one of several mechanisms behind problematic online behavior [9].

Drawing on the literature on the motives for using SM, several studies have pointed to the association between the use of these platforms to regulate unpleasant emotions and the social connection with greater psychological distress [31, 32]. However, notably, there are few significant associations between different motives for use and psychological well-being and distress [32]. The discrepancy with our results may suggest that the structure and focus of the Plan-net 25 scale, by specifically measuring the overvaluation of SM, capture dimensions of SM use that go beyond merely functional or adaptive reasons for use. The Plan-net 25 scale delves into how SM may be perceived as indispensable, thus fostering a psychological dependence that could be a precursor to deterioration in emotional and social well-being.

Theoretically, the tendency to overvalue SM, whether for emotional regulation or social connection, could initiate a detrimental cycle, contributing to the development of PSMU. This dynamic could, in turn, exacerbate and perpetuate psychological distress over time [28, 88]. However, this mechanism is not limited to only these utility domains (i.e., emotional regulation and social connection). According to our results, placing excessive value on SM for purposes such as entertainment or gaining social acceptance can also contribute to this problem. However, these pathways have received less attention in the literature.

In the network model, ORUSM for the regulation of unpleasant emotions was correlated with anxiety but not with depression. This suggests that individuals may overrely on SM to manage their emotions, increasing their vulnerability to anxiety [89]. SM provides short-term relief from anxiety but may reinforce avoidance behaviors over time, preventing the resolution of underlying issues [90]. Thus, anxiety could be a risk factor for PSMU through ORUSM for the regulation of unpleasant emotions. On the other hand, depression, while not linked to ORUSM, is associated directly with PSMU. This may indicate that depression contributes to higher PSMU scores, either through self-critical evaluations [44] or that PSMU leads to increased negative social comparison, increased feelings of inadequacy and worsening depressive symptoms [91]. These results highlight different roles for anxiety and depression in relation to PSMU, suggesting the need for further research (e.g., longitudinal studies).

It is also crucial to highlight the positive link between ORUSM for entertainment and ORUSM for social expression and PSMU. Stockdale and Coyne [38] suggested that the use of SM to avoid boredom was closely related to PSMU, which could imply a parallel finding associating ORUSM and entertainment. Such use may detract from engaging in well-being-enhancing activities such as physical exercise or offline social interactions, thus implying adverse psychological consequences [13, 92]. The subsequent link between ORUSM for social expression and PSMU highlights how people with limited opportunities for social interaction might turn to SM use, increasing the risk of isolation and PSMU [28, 93].

Furthermore, ORUSM for keeping up with current events is positively associated with life satisfaction, even after controlling for other variables, with participants scoring especially high in this domain. It is important to note that the scale does not measure the motivation to use SM for staying informed, but rather the overvaluation of these platforms as a means to achieve that goal. Surprisingly, results show that an elevated valuation in this domain is not linked to negative correlates (e.g., depression, loneliness, low self-esteem, or anxiety). In fact, the positive correlation could suggest that ORUSM for staying informed might even support life satisfaction in adolescents—possibly by reinforcing a sense of community and larger social networks [94]. For instance, remaining digitally connected with friends and family can foster a sense of belonging and enhance well-being [31]. However, the nature of the information to which users are exposed may play a critical role in this dynamic [91].

Moreover, the positive connection between ORUSM for acceptance and loneliness reported in the literature has been replicated [27]. This association could imply that those seeking to alleviate loneliness solely through SM may feel lonelier, potentially because of the superficial nature of online social interactions [49].

Additionally, the minimal relationships between the psychological variables (i.e., distress and well-being) and the Plan-net 25 subscales, after adjusting for model variables, are notable. Like our findings, Groen et al. [95] reported that while there is a link between depression and addiction, their connection weakens in network analyses, a pattern that is consistent with our results.

Finally, although this research focuses on ORUSM, it is important to avoid a narrow focus on a single aspect of problematic online behaviors. The observed comorbidity between different forms of problematic online behavior supports the spectrum hypothesis. This hypothesis suggests that there are distinct yet internet-related constructs, such as social networking, gambling, or online gaming [96, 97]. For example, an ORUSM centered on emotional regulation may act as a gateway, escalating into problematic patterns in contexts such as online gaming or cybersex—behaviors that may share underlying cognitive and emotional processes, leading to excessive behaviors [98]. This may explain behavioral shifts following enforced withdrawal in online activities [99] and highlights the need for a nuanced approach to understanding the multifaceted impact of ORUSM within the broader spectrum of problematic online behaviors (e.g., sexual activities, gaming, or cyberchondria).

### Limitations

This study has several limitations that must be considered. First, achieving representativeness in the expert sample proved challenging. Although efforts were made to include experts of different genders and from diverse Spanish-speaking regions, the sample remained predominantly male and consisted largely of experts from Spain. This may limit the generalizability of the expert consensus obtained to other cultural or regional contexts. Second, the evaluation of the scale’s comprehensibility through cognitive interviews involved two separate groups, one with eight participants and another with only two. While these group interviews provided valuable insights into item clarity, a larger sample size in the second group would have strengthened the robustness of this phase. Third, the sample used to validate the Plan-net-25 scale consisted exclusively of Spanish students aged 12 to 20 years, all attending school in Valencia or Madrid. This narrow demographic focus limits the external validity of the results and warrants caution in generalizing findings to Spanish adolescents as a whole.

### Future lines

Future studies could adapt the Plan-net 25 scale to young adult populations and expand its application across different languages (e.g., French or English) and cultural contexts within the Spanish-speaking world (e.g., Spanish-speaking countries in South America). They can also investigate its psychometric properties, including but not limited to, test‒retest reliability, predictive validity, and future replications of its latent structure. Finally, efforts should focus on comparing this scale with established criteria for measuring SM overvaluation (e.g [20]).,.

## Conclusion

The findings of this study provide promising evidence for the psychometric properties of the Plan-net-25 scale as a tool for assessing ORUSM in Spanish adolescents. By focusing on specific ORUSM domains, such as emotional regulation, social acceptance, and entertainment, this scale offers a more nuanced approach compared to broader measures of SM motives. In clinical contexts, understanding ORUSM across its domains may support the development of targeted interventions that encourage adaptive behaviors as alternatives to PSMU (e.g., offering strategies for emotion regulation without reliance on SM).

## Electronic supplementary material

Below is the link to the electronic supplementary material.

Supplementary Material 1

## Data Availability

The data used and/or analyzed during the current study are publicly available in the “Validation study” folder at the following link: https://osf.io/wc4ev/?view_only=721ad5a81af944d886d82a1ab742f805.

## Abbreviations

AIC: Akaike information criterion
BIC: Bayesian information criterion
CFI: Comparative fit index
CFA: Confirmatory factor analyses
I-CVI: Content validity index
S-CVI: Content validity index for scale
EGA: Exploratory graph analysis
EBIC: Extended Bayesian information criterion
LASSO: Graphical least absolute shrinkage and selection operator
JCR: Journal Citation Reports
MLR: Maximum likelihood estimation with robust standard errors
ORUSM: Overvaluation of the relative utility of social media
PSMU: Problematic social media use
RMSEA: Root mean square error of approximation
SABIC: Sample-size adjusted Bayesian information criterion
SM: Social media
SRMR: Standardized root mean square residual
TLI: Tucker‒Lewis index
