# Supplementary material for: The seven deadly sins: measuring overvaluation of social media with the Plan-net 25 scale
Source: BMC Psychol. 2025 May 27;13:569. doi: 10.1186/s40359-025-02801-1 (PMC12117930; doi:10.1186/s40359-025-02801-1)
Supplement: Supplementary file 1 — Supplementary Material 1 [file 40359_2025_2801_MOESM1_ESM.docx]

**SUPPLEMENTARY MATERIAL**

**Plan-net 25 scale (Original version, validated)**

Las redes sociales son aplicaciones como Instagram, TikTok, WhatsApp, Discord, Twitter, BeReal, etc. que se utilizan para comunicarse y compartir información. En este cuestionario, te presentaremos una serie de afirmaciones sobre las redes sociales.

Imagina por un momento que no puedes acceder a ninguna red social. Nos gustaría que indicaras si, en ese caso, experimentarías dificultades para hacer las cosas que te proponemos a continuación (p.ej., conocer a gente nueva).

No hay respuestas correctas o incorrectas. Tu honestidad y sinceridad al responder las preguntas son fundamentales para obtener resultados precisos.

1: Completamente en desacuerdo; 2: En desacuerdo; 3: Algo de acuerdo; 4: Algo de acuerdo; 5: De acuerdo; 6: Completamente de acuerdo.

| **Si no tuviera acceso a las redes sociales,** | 0 | 1 | 2 | 3 | 4 | 5 |
| --- | --- | --- | --- | --- | --- | --- |
| Tendría muchas dificultades para comunicarme con personas de mi edad. |  |  |  |  |  |  |
| Tendría muchas dificultades para hablar con mis amigos/as. |  |  |  |  |  |  |
| Tendría muchas dificultades para mantener el contacto con mis compañeros/as de clase. |  |  |  |  |  |  |
| Tendría muchas dificultades para quedar con mis amigos/as. |  |  |  |  |  |  |

| **Si no tuviera acceso a las redes sociales,** | 0 | 1 | 2 | 3 | 4 | 5 |
| --- | --- | --- | --- | --- | --- | --- |
| Tendría muchas dificultades para hacer nuevas amistades. |  |  |  |  |  |  |
| Tendría muchas dificultades para encontrar personas con intereses similares a los míos (por ejemplo, hobbies, música, deportes…). |  |  |  |  |  |  |
| Tendría muchas dificultades para conocer a gente nueva (por ejemplo, pareja, amigos/as…). |  |  |  |  |  |  |

| **Si no tuviera acceso a las redes sociales,** | 0 | 1 | 2 | 3 | 4 | 5 |
| --- | --- | --- | --- | --- | --- | --- |
| Tendría muchas dificultades para reducir mi estrés. |  |  |  |  |  |  |
| Tendría muchas dificultades para sentirme mejor cuando estoy triste. |  |  |  |  |  |  |
| Tendría muchas dificultades para calmarme cuando estoy nervioso/a. |  |  |  |  |  |  |
| Tendría muchas dificultades para calmarme cuando estoy agobiado/a. |  |  |  |  |  |  |

| **Si no tuviera acceso a las redes sociales,** | 0 | 1 | 2 | 3 | 4 | 5 |
| --- | --- | --- | --- | --- | --- | --- |
| Tendría muchas dificultades para sentirme incluido/a en mi grupo de amigos/as. |  |  |  |  |  |  |
| Tendría muchas dificultades para sentirme parte de mi grupo de amigos/as. |  |  |  |  |  |  |
| Tendría muchas dificultades para sentirme conectado/a con mis grupos sociales (por ejemplo, amigos/as, familia…). |  |  |  |  |  |  |

| **Si no tuviera acceso a las redes sociales,** | 0 | 1 | 2 | 3 | 4 | 5 |
| --- | --- | --- | --- | --- | --- | --- |
| Tendría muchas dificultades para mantenerme al día con las noticias y eventos actuales. |  |  |  |  |  |  |
| Tendría muchas dificultades para enterarme de lo que hacen las personas de mi entorno. |  |  |  |  |  |  |
| Tendría muchas dificultades para enterarme de lo que hacen los demás (por ejemplo, amigos/as, compañeros/as, familiares…). |  |  |  |  |  |  |
| Tendría muchas dificultades para estar al tanto de lo que sucede en el mundo. |  |  |  |  |  |  |

| **Si no tuviera acceso a las redes sociales,** | 0 | 1 | 2 | 3 | 4 | 5 |
| --- | --- | --- | --- | --- | --- | --- |
| Tendría muchas dificultades para mostrar mis intereses a otras personas (por ejemplo, amigos/as, compañeros/as, familiares…). |  |  |  |  |  |  |
| Tendría muchas dificultades para expresar mis sentimientos. |  |  |  |  |  |  |
| Tendría muchas dificultades para expresar mis pensamientos. |  |  |  |  |  |  |
| Tendría muchas dificultades para dar mi opinión sobre un tema. |  |  |  |  |  |  |

| **Si no tuviera acceso a las redes sociales,** | 0 | 1 | 2 | 3 | 4 | 5 |
| --- | --- | --- | --- | --- | --- | --- |
| Tendría muchas dificultades para entretenerme. |  |  |  |  |  |  |
| Tendría muchas dificultades para pasar el rato. |  |  |  |  |  |  |
| Tendría muchas dificultades para divertirme. |  |  |  |  |  |  |

Para corregir el cuestionario se debe de calcular la media aritmética de cada dimensión. La primera tabla hace referencia al cálculo anormal de la utilidad de las redes sociales para comunicarse con sus iguales, la segunda de conocer a nuevas personas, la tercera de regular emociones desagradables, la cuarta de sentirse incluido/a en su grupo de iguales, la quinta enterarse de lo que está pasando, la sexta expresarse socialmente y la última del entretenimiento.

**Plan-net 25 scale (Translated version, not validated)**

Social media refers to apps such as Instagram, TikTok, WhatsApp, Discord, Twitter, BeReal, etc., which are used to communicate and share information. In this questionnaire, we will present a series of statements about social media.

Imagine for a moment that you cannot access any social media. We would like you to indicate whether, in that case, you would experience difficulties doing the activities listed below (e.g., meeting new people).

There are no right or wrong answers. Your honesty and sincerity when answering the questions are essential to obtain accurate results.

1: Strongly disagree; 2: Disagree; 3: Somewhat disagree; 4: Somewhat agree; 5: Agree; 6: Strongly agree.

| **If I didn’t have social media,** | 0 | 1 | 2 | 3 | 4 | 5 |
| --- | --- | --- | --- | --- | --- | --- |
| I would have a lot of difficulty communicating with people my age |  |  |  |  |  |  |
| I would have a lot of difficulty chatting with my friends. |  |  |  |  |  |  |
| I would have a lot of difficulty staying in touch with my classmates |  |  |  |  |  |  |
| I would have a lot of difficulty meeting up with my friends |  |  |  |  |  |  |

| **If I didn’t have social media,** | 0 | 1 | 2 | 3 | 4 | 5 |
| --- | --- | --- | --- | --- | --- | --- |
| I would have a lot of difficulty making new friends |  |  |  |  |  |  |
| I would have a lot of difficulty finding people with similar interests (e.g., hobbies, music, sports…). |  |  |  |  |  |  |
| I would have a lot of difficulty meeting new people (e.g., partners, friends, etc.). |  |  |  |  |  |  |

| **If I didn’t have social media,** | 0 | 1 | 2 | 3 | 4 | 5 |
| --- | --- | --- | --- | --- | --- | --- |
| I would have a lot of difficulty reducing my stress levels. |  |  |  |  |  |  |
| I would have a lot of difficulty feeling better when I am sad. |  |  |  |  |  |  |
| I would have a lot of difficulty calming myself down when I feel nervous. |  |  |  |  |  |  |
| I would have a lot of difficulty easing my mind when I am overwhelmed. |  |  |  |  |  |  |

| **If I didn’t have social media,** | 0 | 1 | 2 | 3 | 4 | 5 |
| --- | --- | --- | --- | --- | --- | --- |
| I would have a lot of difficulty feeling included in my group of friends. |  |  |  |  |  |  |
| I would have a lot of difficulty feeling like a part of my group of friends. |  |  |  |  |  |  |
| I would have a lot of difficulty feeling connected to my social circles (e.g., friends, family, etc.). |  |  |  |  |  |  |

| **If I didn’t have social media,** | 0 | 1 | 2 | 3 | 4 | 5 |
| --- | --- | --- | --- | --- | --- | --- |
| I would have a lot of difficulty staying updated with current news and events. |  |  |  |  |  |  |
| I would have a lot of difficulty finding out what people around me are doing. |  |  |  |  |  |  |
| I would have a lot of difficulty learning about what others (e.g., friends, colleagues, family members, etc.) are doing. |  |  |  |  |  |  |
| I would have a lot of difficulty keeping up with what is going on in the world. |  |  |  |  |  |  |

| **If I didn’t have social media,** | 0 | 1 | 2 | 3 | 4 | 5 |
| --- | --- | --- | --- | --- | --- | --- |
| I would have a lot of difficulty sharing my interests with others (e.g., friends, colleagues, family members, etc.). |  |  |  |  |  |  |
| I would have a lot of difficulty expressing my emotions. |  |  |  |  |  |  |
| I would have a lot of difficulty expressing my thoughts. |  |  |  |  |  |  |
| I would have a lot of difficulty giving my opinion on a topic. |  |  |  |  |  |  |

| **If I didn’t have social media,** | 0 | 1 | 2 | 3 | 4 | 5 |
| --- | --- | --- | --- | --- | --- | --- |
| I would have a lot of difficulty keeping myself entertained. |  |  |  |  |  |  |
| I would have a lot of difficulty hanging out. |  |  |  |  |  |  |
| I would have a lot of difficulty having fun. |  |  |  |  |  |  |

Scoring instructions: To score the questionnaire, calculate the arithmetic mean of each dimension. The first block refers to the perceived necessity of social media for peer communication. The second relates to meeting new people. The third concerns regulating unpleasant emotions. The fourth refers to feeling included in one's social group. The fifth assesses staying informed about what’s happening. The sixth is about social self-expression. The final one focuses on entertainment.

In order to develop the Plan-net 25 scale, an initial qualitative study was conducted [1]. This study focused on assessing, through four focus groups composed of 26 adolescents, the defining characteristics of PSMU, as well as the utility domains of SM overvalued by this age group. Participants identified different utility domains (or motivations to use SM), including emotional regulation, entertainment, and the use of social media to communicate with their peers. Consistent with our theoretical framework, we proposed that individuals with a limited repertoire of alternative behaviors to achieve specific goals, such as those mentioned, would tend toward more problematic use of these platforms (see [2]). This tendency was also anticipated in subjects who, despite having other options for social interaction, chose to rely solely on social media for this purpose. Thus, it was posited that individuals who exhibit an abnormal computation of the relative utility domains of social media would display problematic usage patterns. Given the scarcity of instruments in the scientific literature to assess this specific construct within the context of social media use, it was decided to develop a psychometric instrument that encompassed the utility domains identified in the focus groups, complementing with previous findings in the literature [3,2,4,5]. It is noteworthy the assistance provided by Chat GPT 3.5 in item generation, demonstrating effectiveness after understanding the underlying theory and measurement model of the construct. This tool generated 200 initial items, these were refined by the research team, followed by consultation with experts from various fields to ensure greater content validity of the questionnaire. Thus, a Delphi study was carried to explore the content validity of the questionnaire. Expert collaboration was not required for utility domain identification or initial item generation [6]. This process was conducted iteratively, allowing experts the opportunity to refine their scores between rounds if deemed necessary [7]. Although prior work recommends the implementation of focus groups, this was not carried out in this study due to logistical difficulties.

Inclusion criteria were set to meet at least one of the following characteristics: (1) Having two years of clinical practice in addiction with or without substance, (2) Having at least two JCR-indexed articles as the first author on behavioral addictions or (3) on methodological studies in psychometric scales. Via email, 25 experts were invited, with 14 participating in the first round (56%).

Figure S1 illustrates the geographical distribution of the experts, with a higher representation in the Valencian Community, Andalusia, and the Community of Madrid in Spain. There was also one participant in Belgium (Louvain) and another in Australia (Melbourne). All participants were Spanish speakers.

Figure S1.

Maps of Spain, Australia, and Belgium showing the provinces of origin for each country.


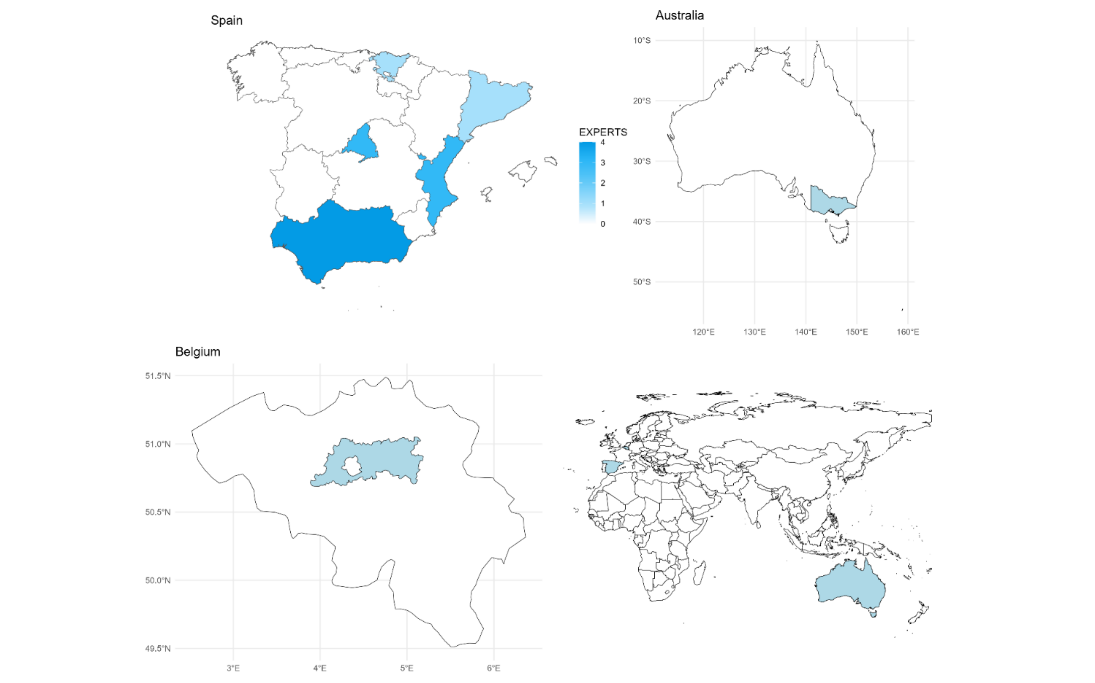


Table S1 provides a detailed overview of the socio-demographic profile of the experts who participated in the study. Among them, 46.15% have experience in addiction clinics, while 53.84% have experience in clinical intervention with adolescents. Notably, 38.46% of the participants report experience in both addiction clinics and adolescent care, highlighting a mixed professional profile.

Table S1

Socio-demographic data of the expert panel

| Expert | Workplace | City | Country | Q1 | Q2 | Q3 |
| --- | --- | --- | --- | --- | --- | --- |
| Juan Manuel Machimbarrena | University of the Basque Country | Bilbao | Spain | No | No | No |
| Alex Palau | Child and Adolescent Mental Health Center - Sant Joan de Déu Terres de Lleida | Lleida | Spain | Yes | Yes | No |
| Juan Francisco Navas | Complutense University of Madrid | Madrid | Spain | Yes | Yes | No |
| Oscar Lecuona | Autonomous University of Madrid | Madrid | Spain | No | No | Yes |
| Jesús Castro | University of Valencia | Valencia | Spain | Yes | No | No |
| Inés Tomás | University of Valencia | Valencia | Spain | No | No | Yes |
| Irene Checa | University of Valencia | Valencia | Spain | Yes | Yes | Yes |
| Silvana Romero | Catholic University of Louvain | Louvain | Belgium | Yes | Yes | Yes |
| Daniel Zarate | RMIT University | Melbourne | Australia | No | Yes | Yes |
| Sergio Fernández | Loyola University | Sevilla | Spain | Yes | Yes | No |
| Ismael Muela | University of Granada | Granada | Spain | No | No | No |
| Olatz López | Complutense University of Madrid | Madrid | Spain | No | Yes | No |
| José César Perales | University of Granada | Granada | Spain | No | No | No |
| Óscar Lozano | Huelva University | Huelva | Spain | No | No | Yes |
| Note. Q1 = Two years of clinical practice in behavioural or substance addiction; Q2 = Two years of clinical practice with adolescents; Q3 = Expertise in methodology. | | | | | | |

After confirming their participation, 14 experts received a link through the Qualtrics platform to proceed with the evaluation of the preliminary scale. This platform was used to define the central construct of the study: ORUSM, explaining its main components based on previous literature. Experts were provided with detailed guidance on how to assess each of the initial items regarding three specific criteria: Clarity, Appropriateness, and Relevance.

Clarity evaluated the ease of understanding of each item, ensuring that it was interpretable without ambiguities and free of confusion. The rating scale ranged from 1 (not clear at all) to 4 (very clear).

Appropriateness referred to how well the item represented the specific dimension it intended to measure within the construct, using a rating scale from 1 (not appropriate at all) to 4 (very appropriate).

Relevance measured the importance of the concept each item attempted to evaluate within the context of the study, using a scale from 1 (not relevant at all) to 4 (very relevant).

Subsequently, experts were asked for suggestions for the overall improvement of the questionnaire, including modifying existing items or creating new ones. The original instructions provided to the experts, as well as their translation into English, are available in the document "Instructions for the experts.pdf" hosted on OSF. This document reflects how the initial measurement model evolved into a more robust and suitable structure thanks to the contributions of the experts. We do not recommend using the English version yet, as a thorough translation of the questionnaire has not been completed.

For this first round of evaluation, 55 items were generated and grouped into 6 different utility domains, corresponding to the potential ORUSM factors identified preliminarily. The suitability of the items and the scale as a whole were evaluated using two indices: the Content Validity Index for items (I-CVI) and the Content Validity Index for scales (S-CVI), applying the established calculation method [8].

The I-CVI was calculated as the proportion of experts who assigned values of 3 or 4 to an item, divided by the total number of participating experts. The S-CVI was obtained by averaging the I-CVI values of all items within an utility domain (e.g., each item would have an I-CVI value for clarity, and the scale would have a clarity value called S-CVI). Following Lynn's recommendations [10], a cutoff point of .78 was adopted, although other values have been suggested in the literature (e.g., .80 [9]).

Despite the results of the first round showing S-CVI scores of .89 for clarity, .85 for Appropriateness, and .81 for Relevance, we decided to refine the items and proceed to a second round, due to the valuable qualitative information provided by the experts. Thus, all items with an I-CVI below .78 were eliminated [10]. Additionally, based on the feedback received from the various experts, items were either completely removed or expert suggestions were incorporated.

If any reader is interested in the specific scores from the first round for each item and for the scale overall, they can access the following document "Content validity 1st round.pdf" at the following link: [https://osf.io/wc4ev/?view_only=721ad5a81af944d886 d82a1ab742f805](https://osf.io/wc4ev/?view_only=721ad5a81af944d886%20d82a1ab742f805) :

Subsequently, a personalized report was generated for each expert in a .csv document. It showed the value assigned to each item in each dimension by the expert. Additionally, the overall score obtained by the item in the I-CVI was displayed. Since the selected cutoff point was indicated, the experts could identify which items would not continue to the next round due to the cutoff. Finally, there was a column where experts could adjust their initial item ratings in light of the collective results. An example of the feedback received by one of the experts is available on OSF; however, the name of the expert has not been revealed to maintain anonymity ("Feedback received by the experts – Example.xlsx"). None of the experts decided to change their initial ratings based on the results of the other experts.

Based on the suggestions from the experts in the first round, the following changes were implemented:

- Use a common header for all items (saving time for participants).
- Include a new utility domain called "Entertainment”
- Change some items from the utility domain to which they belonged in the first round of evaluation.
- Eliminate the utility domain "Learning and skill development" as it was not highly relevant in the previous focus groups and the items received low scores in I-CVI for Appropriateness and Relevance. Additionally, there was anticipated low clinical predictive capacity for this utility domain.
- Make slight modifications to the questionnaire introduction.

The second and final round of review began with the participation of 13 experts; one of the original experts did not continue in this phase. In total, 54 items were evaluated, including those previously examined in the first round, modifications to pre-existing items, and new items. The inclusion of previously evaluated items was due to changes in the overall structure of the questionnaire, which involved adjustments in wording (creation of a common header). The 6-point scale was proposed instead of the 5-point scale because participants do not adequately reflect their ratings when there is an intermediate option [11].

The results of the second round reflected an increase in the Content Validity Index for the Scale (S-CVI), reaching .91 for clarity, .92 for Appropriateness, and .90 for Relevance. Despite the presence of redundant items, intentionally introduced to assess options with better psychometric properties, the process proceeded to select the most robust items while always maintaining the content validity of the evaluated construct. The reception of the Likert scale by the experts was positive, indicating a favorable acceptance of the improved instrument after incorporating feedback from the first round. For those interested in examining the scores of the second round in detail, the document "Content validity 2nd round.pdf" is available at [https://osf.io/wc4ev/?view_o nly=721ad5a81af944d886d82a1ab742f805](https://osf.io/wc4ev/?view_only=721ad5a81af944d886d82a1ab742f805)

To optimize the practical application of the scale in research and clinical settings, it was decided to reduce the number of items. After eliminating those items with lower and redundant evaluations, a final scale of 27 items distributed across 7 utility domains was formed. This version underwent a pilot study with 17 adolescents recruited through social media and personal contacts of the researchers. Participants provided informed consent, sociodemographic data, and responded to the preliminary version of the questionnaire. Subsequently, they assessed the clarity of the items using a 3-point categorical scale. They also provided qualitative feedback on the understanding of the questionnaire and possibilities for improvement.

Preliminary results indicated the need for minor adjustments to some items. The document "Pilot study items.pdf" on OSF details the quantitative feedback received from the adolescents.

Consequently, two group cognitive interviews with adolescents were organized. This method involves conducting detailed interviews with participants after they have completed a questionnaire, with the aim of understanding how they interpreted the questions, what cognitive processes they used to answer them, and how they formulated their responses.

Although the sample size was small (n = 10), the methodology employed aligned with typical focus group methodologies, fostering interaction and exchange of perspectives among participants. This stage of the study provided valuable insights for the simplification and improvement of specific items, thus enriching the understanding of the measured construct and the applicability of the instrument. To do so, the recommendations of Padilla and Benítez [12] were followed. Hence, two sessions of semi-structured interviews were conducted, the first face-to-face with 8 participants and the second online with 2 participants, due to logistical and time constraints. The participants, 7 females and 3 males aged between 14 and 17 years old, answered the Social Media Disorder Scale [13], sociodemographic data and the Plan-net 25 scale. The sociodemographic data of the participants can be found in the document "Validity based on response processes. Descriptives.pdf". Subsequently, the think-aloud protocol was used during cognitive interviews to explore the psychological and cognitive processes when answering the questionnaire. This approach allowed for discussions on the understanding of complex terms, instructions, and the Likert scale used.

Analysis of the cognitive interviews revealed several key insights regarding the comprehension of items and the questionnaire's structure. Participants expressed difficulties with complex expressions and specific terms, suggesting the need for adjustments in the wording of some items to improve clarity and ease of response. The second interview session was particularly valuable for confirming the initial interpretations and improvement suggestions.

Based on these findings, adjustments were made to the items to address the identified difficulties, including simplifying terms and clarifying instructions. These changes were implemented to optimize the questionnaire and ensure its suitability for the target population. The final version of the questionnaire is included in the document "Plan-net 25 scale.pdf". Additionally, caution is emphasized when using the automatically translated version of the questionnaire, given the lack of formal validation of the translation.

**A priori determination of sample size**

After deciding the final number of items of the questionnaire and the number of factors, the necessary sample size was calculated to accurately analyze the study's parameters, such as the factor loadings of the items and the covariances among latent factors, ensuring a statistical power of at least .80. Using the ShinyApp pwrSEM, developed by Wang and Rhemtulla [14], the factor model was simulated. The validation results of two scales related to the motives for using SM, considering their possible similarity, were used as input for the simulation [4,5]. After setting a sample size of 1,750 participants, a significance level α of .05, and conducting 10,000 Monte Carlo simulations, a power of 1.00 was achieved for all parameters of interest. However, the latent correlation between factor 1 (ORUSM for interacting socially) and factor 6 (ORUSM for expressing oneself socially) had a power of .81, and between factor 6 and 7, a power of .99.

**Exploratory Dimension Reduction**

After completing the data cleaning process, we proceeded with the division of the sample. We randomly selected 800 observations using a seed to ensure the reproducibility of the sampling. Initially, we planned to select only 500 observations. However, since we had already achieved the required sample size for CFA, we decided to increase the sample size for the exploratory analyses, given that it was underpowered to detect the adequate latent structure of the scale. This subset of data was designated for the exploration of the factorial structure of the instrument, applying two different analytical techniques to identify the most robust and adequate factorial configuration of the studied construct. Initially, a parallel analysis was conducted, a technique recommended for determining the number of factors to retain in exploratory factor analysis, providing an empirical basis for this decision. The details and specific results of this analysis are illustrated in Figure S2 of the supplementary material.

Figure S2

Scree plot of parallel analysis for actual and simulated data in Principal Components and Factor Analysis


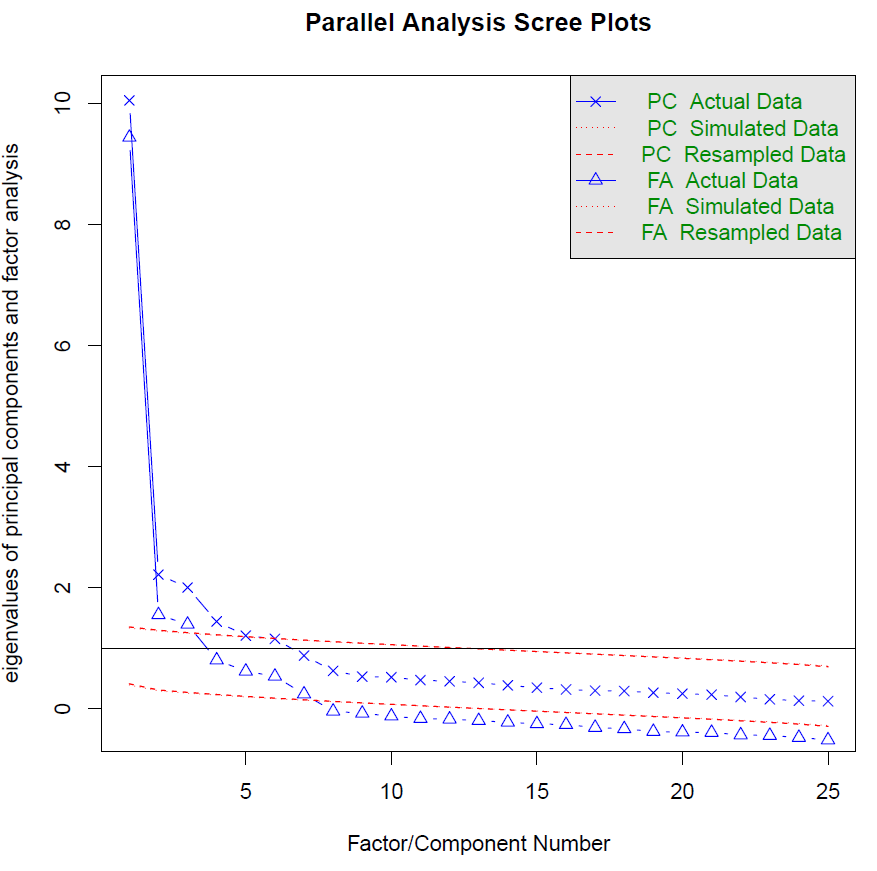


The interpretation of the results indicates that a 7-factor structure adequately represents the studied construct, based on the factorial parallel analysis. Although principal component analysis suggested a more stable five-component solution, the evidence gathered during the refinement of the scale and the findings from response process validation favor a 7-factor structure. This choice better aligns with the underlying theory and the collected qualitative information, highlighting the relevance of combining different methods and evidence in the validation of psychometric instruments.

Additionally, to avoid relying solely on parallel analysis, we opted to implement Exploratory Graph Analysis (EGA). This technique was chosen for its demonstrated superior capacity in simulation studies to detect the latent structure of items, according to Golino & Epskamp [15] and Golino et al. [16]. Thus, we proceeded to conduct EGA. For this purpose, we applied gLASSO (graphical Least Absolute Shrinkage and Selection Operator) regularization to the generated network, following the Extended Bayesian Information Criterion (EBIC) with a default hyperparameter of .50.

To examine the variability of the psychometric network in question, the EGA was conducted twice, each with a different algorithm for community detection: "Louvain" and "Walktrap". These methods facilitate understanding the organization of the network into communities, a feature quantified by modularity. Modularity is a metric that measures the quality of dividing a network into communities, indicating the density of connections between nodes within the same communities compared to those between different communities. A high modularity value denotes a clear division into densely interconnected communities but weakly linked between them. Walktrap algorithm detects communities through random walks, based on the principle that stronger connections increase the probability of two nodes belonging to the same community. On the other hand, Louvain algorithm focuses on maximizing modularity by relocating nodes between communities, seeking the configuration that optimizes the density of intra-community connections contrasted with inter-community connections. This process is repeated until no further increase in modularity is achieved or a one-dimensional structure is reached.

The empirical network of the Plan-net 25 scale, obtained after applying the Louvain method, is presented in Figure S3. This network indicates that the latent structure is composed of 6 factors. Specifically, according to these results, items from the first and second utility domain could cluster together. Consequently, this solution was tested using CFA.

Figure S3

Empirical network of Plan-net 25 scale items using Lovain algorithm.
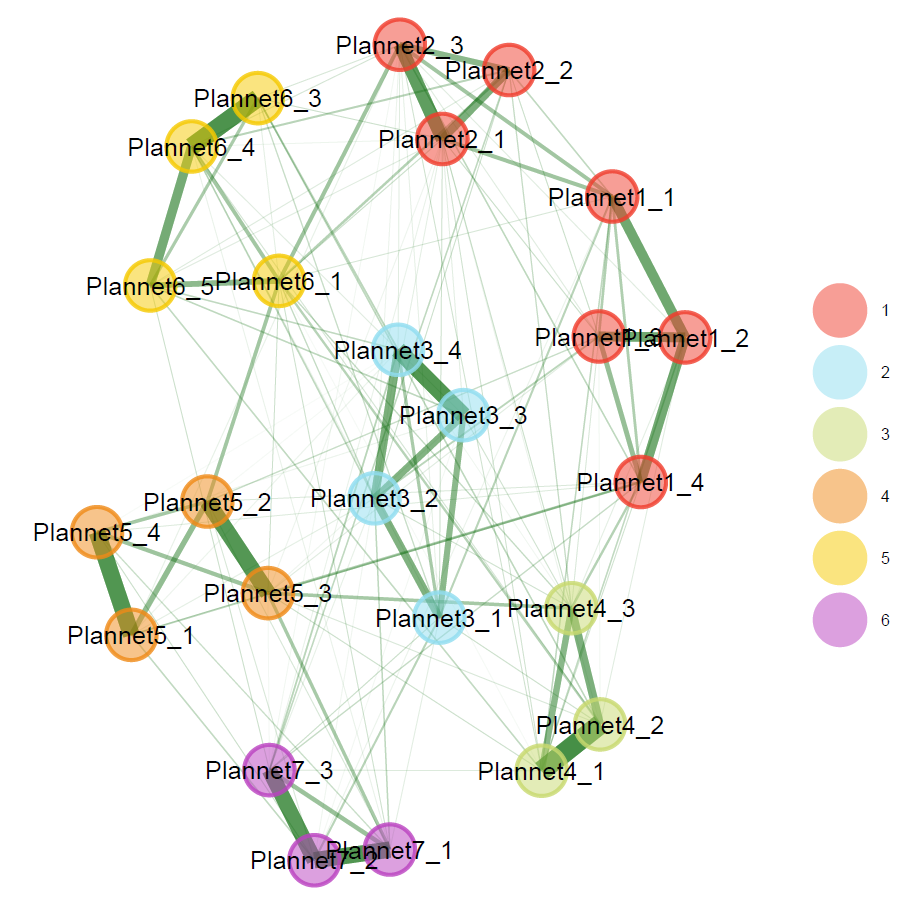


Next, the figure obtained using EGA with Walktrap algorithm is presented to evaluate possible differences in community identification among both networks. Figure S4 displays the empirical network with Walktrap algorithm. Contrary to the former network, this network indicates a latent structure composed of 7 factors, as suggested by the Delphi Study.

Figure S4

Empirical network of Plan-net 25 scale items using Walktrap algorithm.
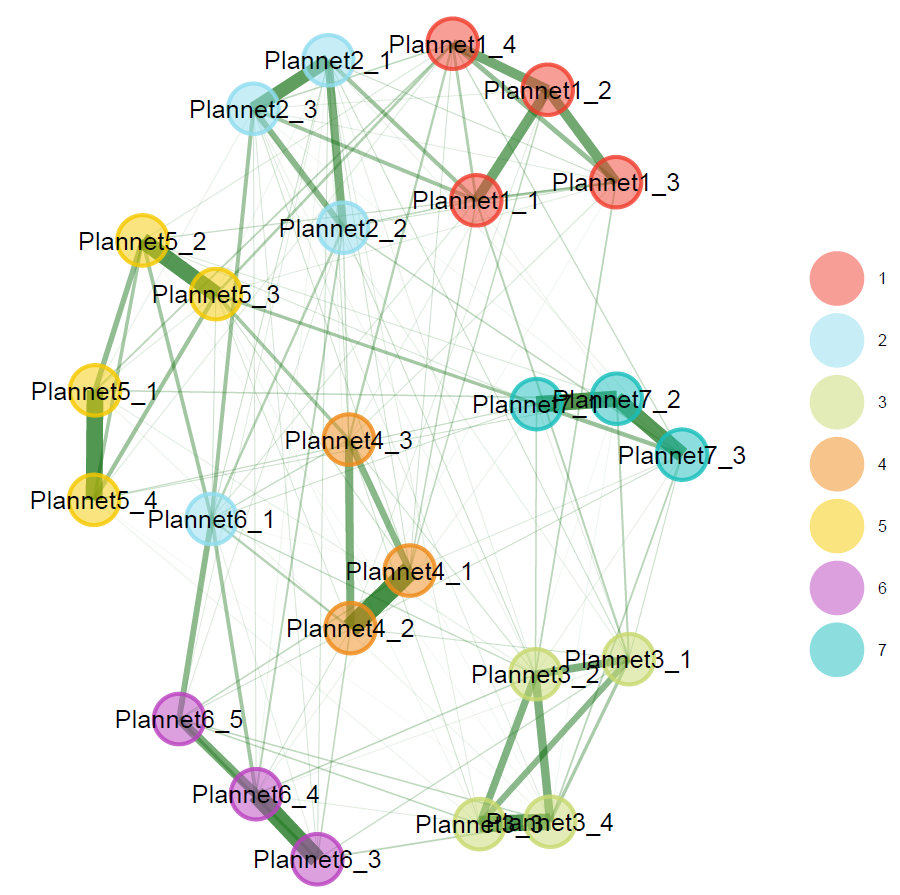


Therefore, based on these exploratory results, the following decision was made. Given that the parallel analysis using principal components suggested a five-factor solution but did not specify which items belonged to each factor, this proposal was discarded. Additionally, the solution that combined the first and second theoretical factors into a single factor was tested using CFA models. Furthermore, the theoretical solution initially proposed in the Delphi study was supported by both EGA using the Walktrap algorithm and parallel analysis using factors.

**Description of the scale**

Frequencies of each response category for each item of the Plan-net 25 are presented below (Figure S5). This facilitates the identification of possible floor and ceiling effects.

Figure S5

Graph depicting the percentage of response for each item of the Plan-net 25 scale.


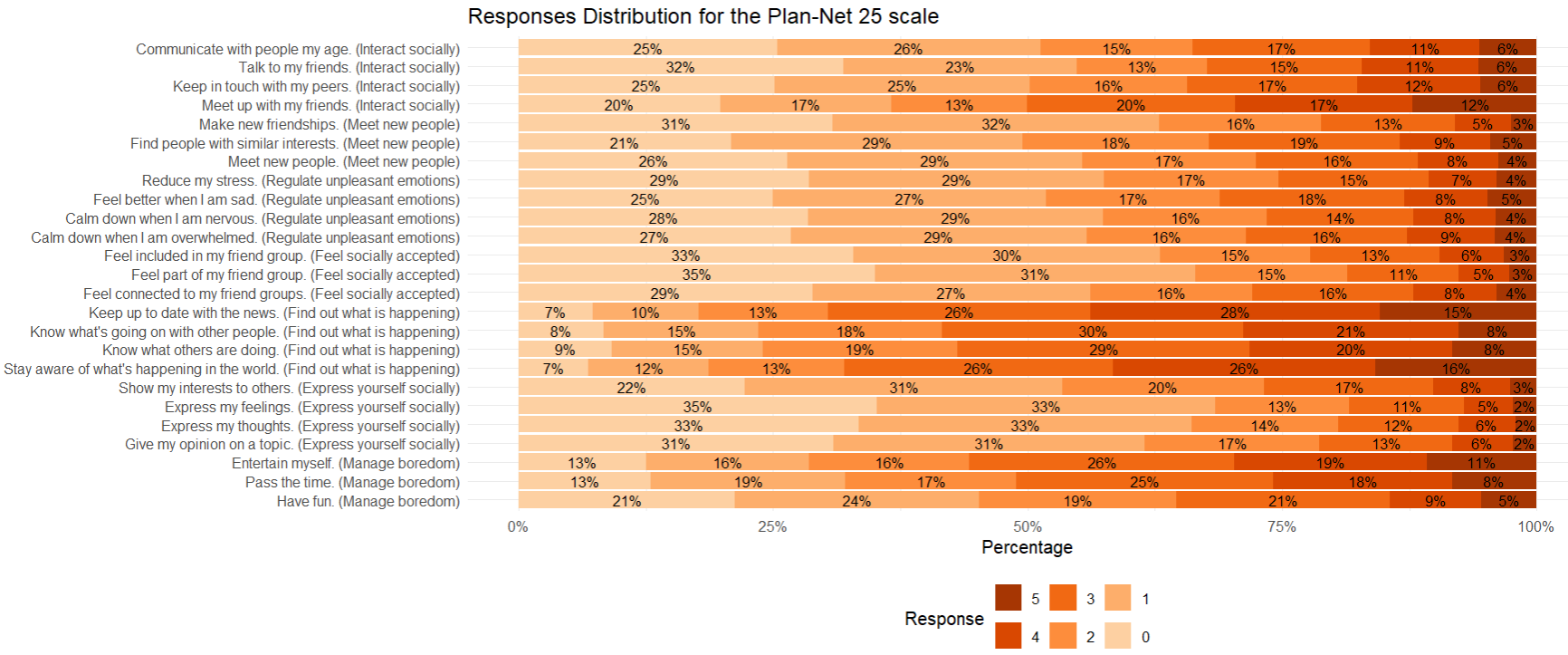


**Correlation among items of the Plan-net 25 scale**

A graph illustrating the Pearson correlations between the items of the Plan-net 25 scale is presented. Rather than directly extracting and listing the correlation values from the matrix, this visual approach has been chosen to facilitate understanding of the relationships between items, both within the same utility domain and across different utility domains.

Figure S6 displays Pearson correlations, where item groupings can be observed based on their correlations. Plannet6_2 is not included because it was an attentional check item.


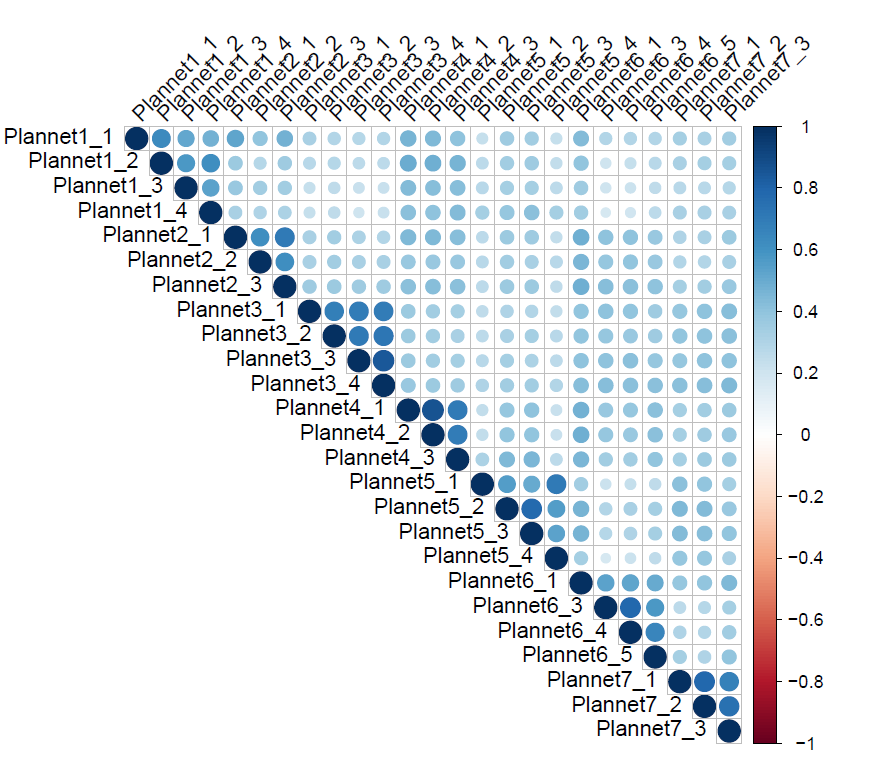


Figure S6. Pearson correlation graph among the items of the Plan-net 25 scale. Blue scores indicate a positive association, while red scores indicate a negative association. The intensity of the color indicates the effect size of the association.

**Correlation among factors**

Figure S7 shows the latent covariances among the 7-factor structure.

Figure S7

Correlation graph among the 7 factors of the Plan-net 25 scale. **
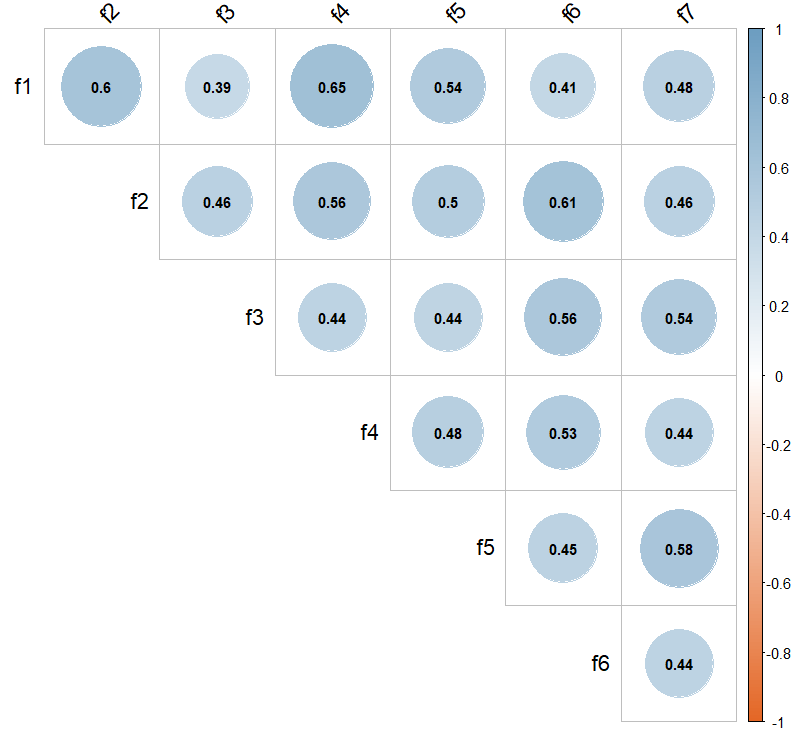
**

Blue scores indicate a positive association, while red scores indicate a negative association. The intensity of the color indicates the effect size of the association. Estimates were obtained from the structural equation model.

**Independent samples t-test among Plan-net 25 subscales**

Given the support for the invariance of latent means between boys-girls and early adolescents-late adolescents, independent sample tests using factor scores were conducted between these groups. The following table shows whether the factor scores of each subscale can be considered statistically different by gender or age. To control the Type I error rate, the Bonferroni correction was applied.

Table S2

T-test comparisons between gender and age groups regarding different Plan-net 25 subscales.

| Independent variable | Dependent Variable | Group 1 | Group 2 | *t* | df | *p* |
| --- | --- | --- | --- | --- | --- | --- |
| Gender | Plan-net1 | 909 | 960 | -5.16 | 1865 | **< .001** |
|  | Plan-net2 | 909 | 960 | -2.83 | 1862 | **.004** |
|  | Plan-net3 | 909 | 960 | -5.12 | 1866 | **< .001** |
|  | Plan-net4 | 909 | 960 | -4.77 | 1866 | **< .001** |
|  | Plan-net5 | 909 | 960 | -3.93 | 1861 | **< .001** |
|  | Plan-net6 | 909 | 960 | -2.98 | 1867 | **.003** |
|  | Plan-net7 | 909 | 960 | -2.75 | 1862 | **.006** |
| Age | Plan-net1 | 1212 | 657 | -3.63 | 1311 | **< .001** |
|  | Plan-net2 | 1212 | 657 | -3.18 | 1303 | **.001** |
|  | Plan-net3 | 1212 | 657 | -1.95 | 1331 | .05 |
|  | Plan-net4 | 1212 | 657 | -1.34 | 1369 | .179 |
|  | Plan-net5 | 1212 | 657 | -5.25 | 1369 | **< .001** |
|  | Plan-net6 | 1212 | 657 | -.670 | 1348 | .50 |
|  | Plan-net7 | 1212 | 657 | -3.62 | 1380 | **< .001** |

Note. Group 1 in Gender = Boys; Group 2 in Gender = Girls; Group 1 in Age = Early adolescents; Group 2 in Age = Late adolescents. Bold values indicate significant differences (p < .05) among the two groups. Plan-net1: ORUSM for interacting socially; Plan-net2: ORUSM for meeting new people; Plan-net3: ORUSM for regulating unpleasant emotions; Plan-net4: ORUSM for feeling socially accepted; Plan-net5: ORUSM for keeping up with what is happening; Plan-net6: ORUSM for expressing oneself socially; Plan-net7: ORUSM for managing boredom

Factor scores mean for each group.

Table S3

Factor scores mean for each group regarding different Plan-net 25 subscales.

| Gender | Plan-net 1 | Plan-net 2 | Plan-net 3 | Plan-net 4 | Plan-net 5 | Plan-net 6 | Plan-net 7 |
| --- | --- | --- | --- | --- | --- | --- | --- |
| Boys | **-.135** | **-.0796** | **-.132** | **-.145** | **-.0843** | **-.0668** | **-.0758** |
| Girls | **.121** | **.0546** | **-.0278** | **.128** | **.0861** | **.0443** | **.0830** |
| Age |  |  |  |  |  |  |  |
| Early adolescents | **-.0709** | **-.0668** | -.0402 | -.0332 | **-.0798** | -.0189 | **-.0702** |
| Late adolescents | **.120** | **.0928** | .0594 | .0473 | **.156** | .00730 | **.146** |

Note. Bold values indicate significant differences (*p* < .05) among the two groups. Plan-net1: ORUSM for interacting socially; Plan-net2: ORUSM for meeting new people; Plan-net3: ORUSM for regulating unpleasant emotions; Plan-net4: ORUSM for feeling socially accepted; Plan-net5: ORUSM for keeping up with what is happening; Plan-net6: ORUSM for expressing oneself socially; Plan-net7: ORUSM for managing boredom

**Confirmatory factor analyses**

Before conducting Pearson’s correlation matrix or network analysis, a confirmatory factor analyses were fitted for each scale. This was done in order to obtain factor scores for each measure. Models were fitted using Maximum Likelihood with Robust Correction and missing values were handled using full information maximum likelihood. All models were unifactorial. Table S4 indicates the results.

Table S4

Confirmatory factor analyses for each scale.

| Model | χ² | df | *p*-value | CFI | TLI | RMSEA | SRMR |
| --- | --- | --- | --- | --- | --- | --- | --- |
| Patient Health Questionnaire-9 | 364.511 | 27 | < .001 | .932 | .911 | .093 [.085, .101] | .039 |
| Generalized Anxiety Disorder-7 | 147.229 | 14 | < .001 | .971 | .957 | .085 [.073, .098] | .026 |
| Satisfaction With Life Scale-3 | - | - | - | - | - | - | - |
| Social Media Disorder Scale-9 | 379.999 | 27 | < .001 | .908 | .887 | .102 [.093, .111] | .046 |
| Three-item Loneliness Scale | - | - | - | - | - | - | - |

Note. Fit indices could not be computed for the 3-item scales because the degrees of freedom were 0, resulting in a saturated model. The PHQ, GAD, and SMD scales did not show excellent psychometric properties based on the reported fit indices. However, the results were evaluated using mean scores and factor scores, and the conclusions remained unchanged.**Network analysis**

As specified in the main manuscript, a network analysis was conducted using factor scores obtained after adjusting the confirmatory factor analysis model for each scale. In this analysis, a set of variables (or items) referred to as nodes were included in a model. Subsequently, the relationships between these nodes were modeled. In this case, partial correlations between these variables were employed, with these relationships termed edges. Due to the cross-sectional design, these partial correlations represented undirected conditional associations. The position of each node was determined using the Fruchterman-Reingold algorithm, which arranges nodes with stronger connections in closer proximity [17]. A polychoric correlation matrix was employed using the "cor_auto" command from the "bootnet" package, which also automatically calculates polyserial or Pearson correlations if necessary [19]. This was done because self-esteem was measured with a single item, making it impossible to compute a factor score for this item.

The graphical Least Absolute Shrinkage and Selection Operator (gLASSO) regularization technique was implemented, guided by the Extended Bayesian Information Criterion (EBIC) with a default hyperparameter adjustment of .50. The gLASSO regularization aims to simplify and clarify the network structure by reducing its complexity through promoting a sparser network: numerous connection weights are shrunk to zero and eliminated, thus highlighting the most significant connections. The selection of the optimal tuning parameter through EBIC ensures a balance between model simplicity and fit [19]. In the network visualization, positive and negative correlations were represented by green and red edges, respectively, where the thickness of the edges indicates the strength of the partial correlations [20].

Figure S8

Network estimated using EBICglasso.


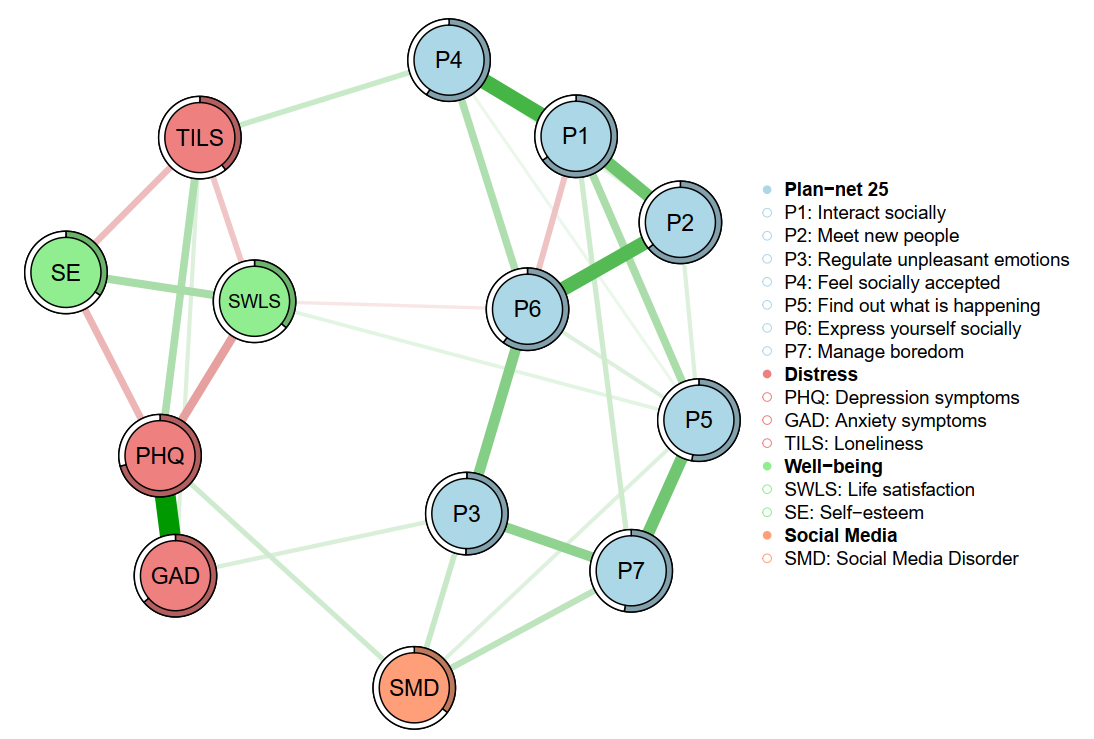


Subsequently, strength, betweenness, and closeness metrics were calculated for each node in this network.

As illustrated in Figure S8, nodes Plan-net 6, Plan-net 1, and PHQ present the highest Strength values, while SMD shows a reduced value. These centrality values should be interpreted alongside the network. In fact, there is a clear separation between two components: on one side are the psychological variables (i.e., depression, anxiety, loneliness, self-esteem, and life satisfaction), and on the other side are the sub-scales of the Plan-net 25 scale. Figure 9 indicates the strength, betweenness and closeness of each node. However, it is necessary to consider the stability and precision of the model to determine if the differences in centrality estimates are significant [19].

Figure S9

Strength, betweenness, and closeness of each node in the network.


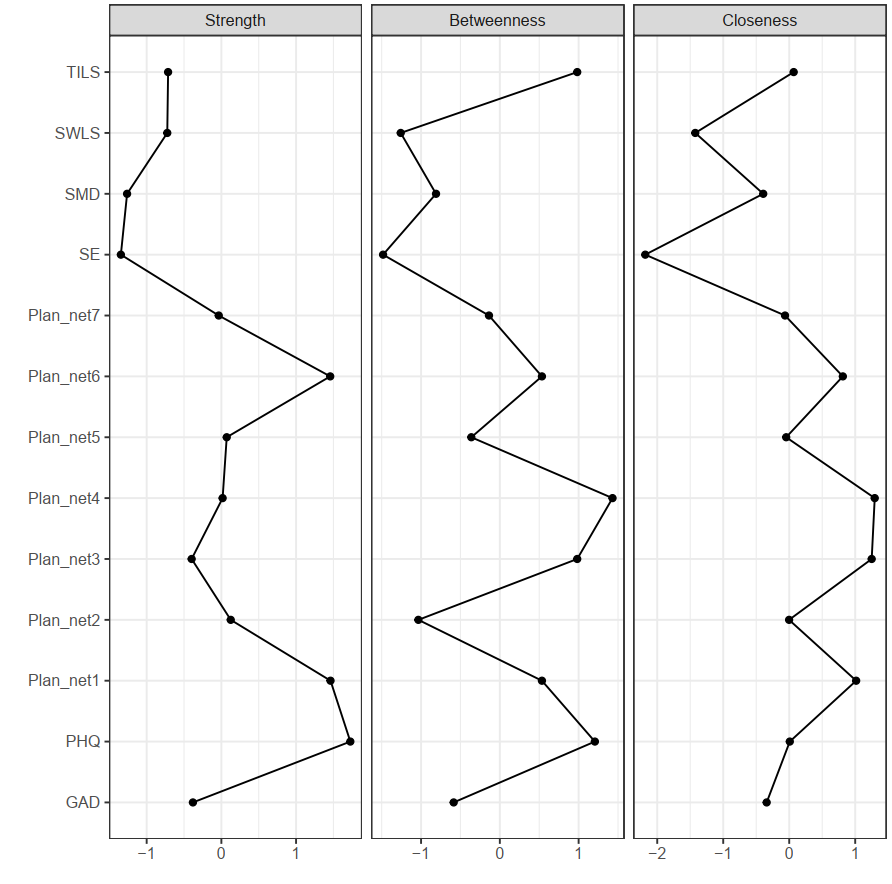


**Stability and Precision Assessment of the Network Model**

The robustness of the psychometric network was evaluated using a non-parametric bootstrap method with 5000 iterations to determine the stability of the network elements. The following figures show this assessment. Specifically, Figure S10 illustrates the bootstrap confidence intervals for the estimated weights of the network connections. The red line indicates the values obtained directly from the sample, while the gray area denotes the confidence intervals derived from the bootstrap. Each horizontal line represents a connection within the network, ordered from highest to lowest weight [19]. It is important to analyze the overlapping confidence intervals in Figure S10. When two intervals overlap, it indicates that there are no statistically significant differences between the weights of those connections.

Figure S10

Connection weights of the estimated network with bootstrap confidence intervals.
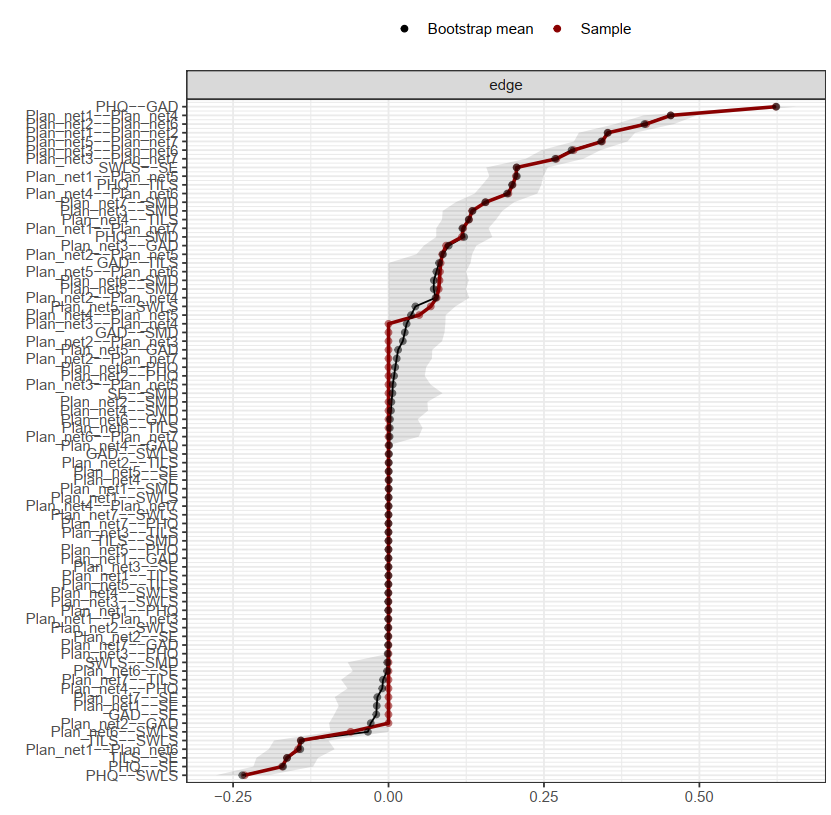


The horizontal axis shows the weights of the connections, with black dots representing bootstrap means and red dots representing observed weights in the sample. Black lines indicate confidence intervals.

Figure S11 highlights significant differences between connections (edges) in the network.

Figure S11

Bootstrap significance test for differences between connection weights.


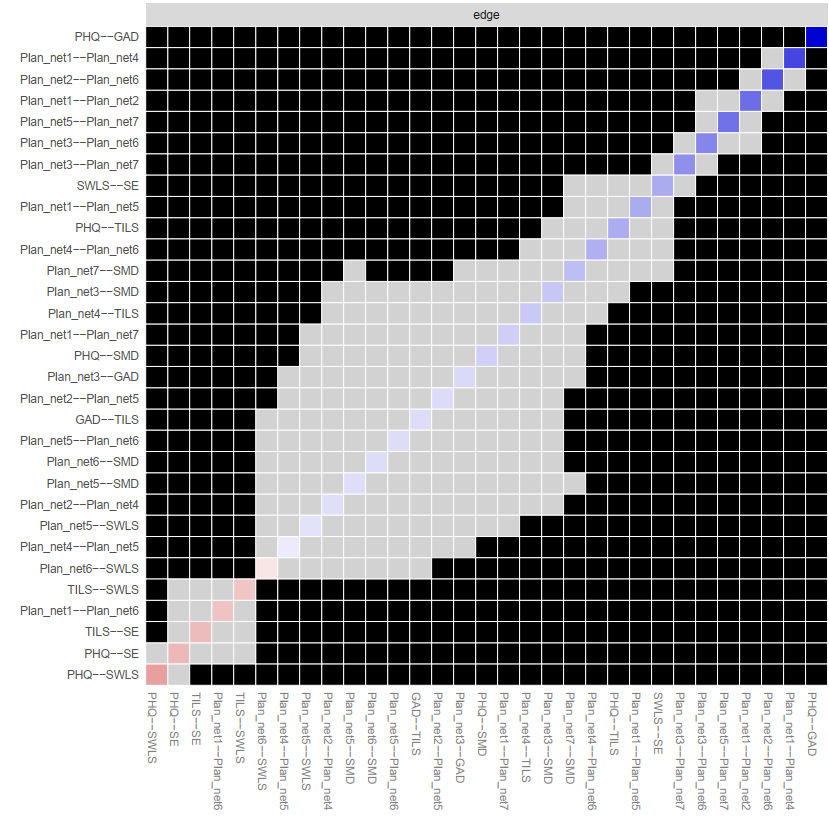


Gray boxes indicate connections or nodes without significant differences, while black boxes indicate those with significant differences. Colored boxes correspond to the colors of the connections in the plot.

Figure S12 displays significant differences in terms of strength between different nodes in the network.

Figure S12.

Bootstrap significance test on node strength differences in the network.


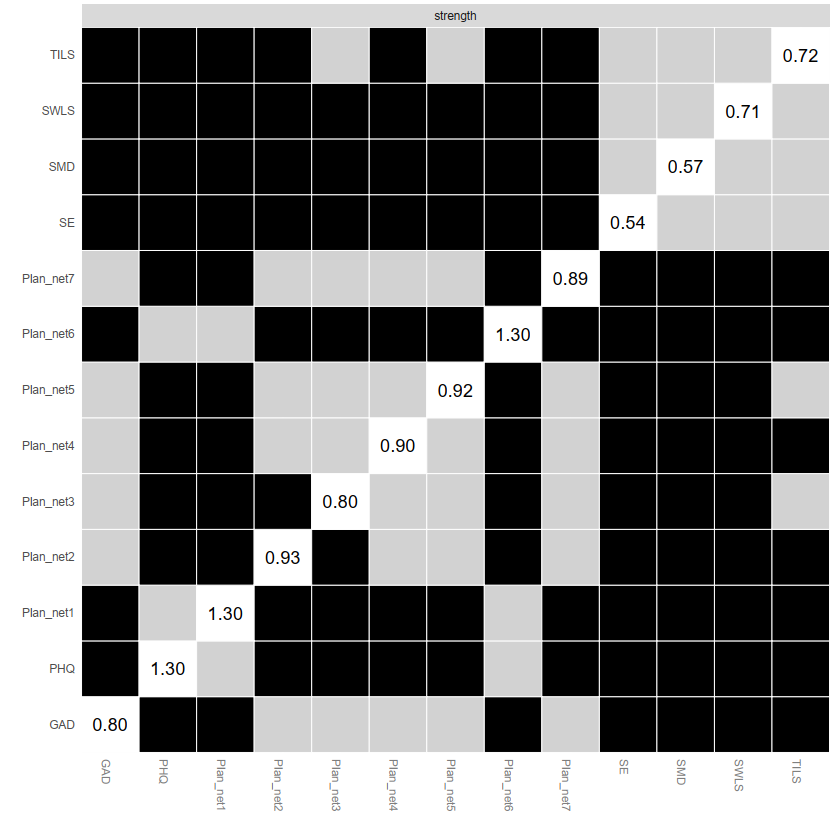


Gray boxes show nodes without significant differences, while black boxes highlight significant differences in node strength. White boxes indicate the value of each node's strength.

Subsequently, a bootstrap analysis with 5000 iterations was conducted to assess the stability of centrality indices, as depicted in Figure S13. The stability coefficient reflects the proportion of the sample that can be excluded while ensuring that the correlation of centrality indices with those derived from bootstrap samples remains at .70 or higher within a 95% confidence interval. Cut-off criteria are considered acceptable above .25 and adequate above .50 [19]. In this study, lower values are observed in the betweenness index, indicating poor stability (CS coefficient = .05). The stability of closeness is even lower, with a CS coefficient of 0, suggesting that any reduction in the sample significantly impacts its stability. In contrast, the expected influence and strength indices show excellent stability, with both achieving a CS coefficient of .75. This surpasses the adequate cutoff point of .50, indicating that these indices remain robust even when a substantial portion of the sample is excluded.

Figure S13

Average correlations of centrality indices with excluded cases compared to the original sample.


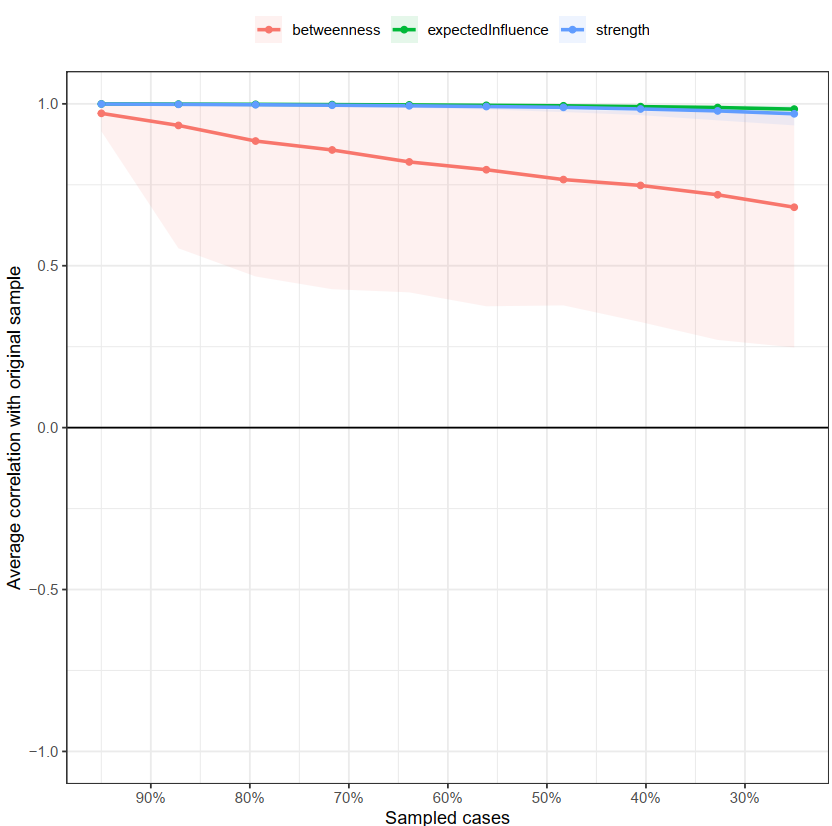


The lines show the means, and the shaded areas represent the ranges from the 2.5th to the 97.5th quantiles.

Finally, a power analysis was conducted for the estimated networks in relation to the actual network, as well as for the sensitivity, specificity, and centrality indices. To observe the change in values as sample sizes are modified, conditions of 500, 1000, 2000, 2500, and 5000 were included. Additionally, the number of participants included in the network was also included (n = 1798). Under this condition, a correlation of .99 was observed between the estimated and actual networks, with a sensitivity of .89 and a specificity of 1, suggesting the adequacy of the achieved sample size (see Figure S14).

Regarding centrality indices, the accuracy of the network was around 97% for strength, approximately 89% for closeness, and around 84% for betweenness (see Figure S15). The fact that the values of betweenness and closeness are not as high is not surprising, as these two centrality indices have been reported as more unstable [21,22]. Nevertheless, they have acceptable values for interpretation given the obtained sample size.

Figure S14.

Simulation Analysis of Correlation, Sensitivity, and Specificity Across Different Sample Sizes in the Psychometric Network


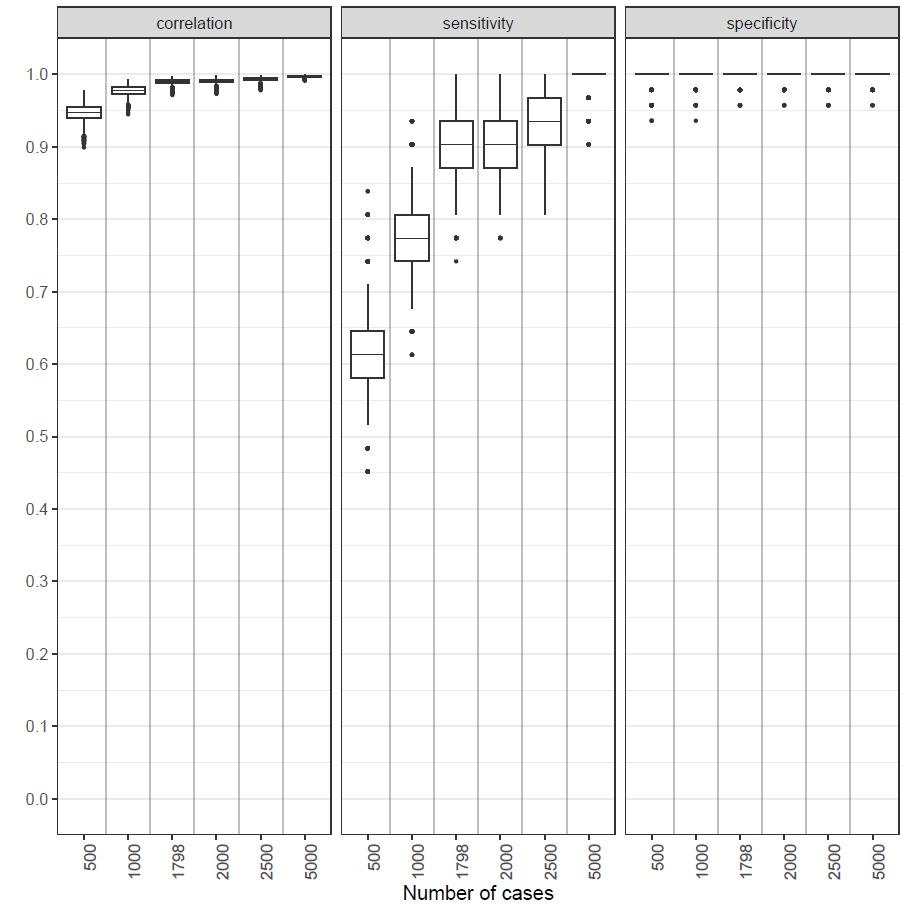


Figure S15.

Simulation Analysis of Centrality Measures in Psychometric Networks at Different Sample Sizes


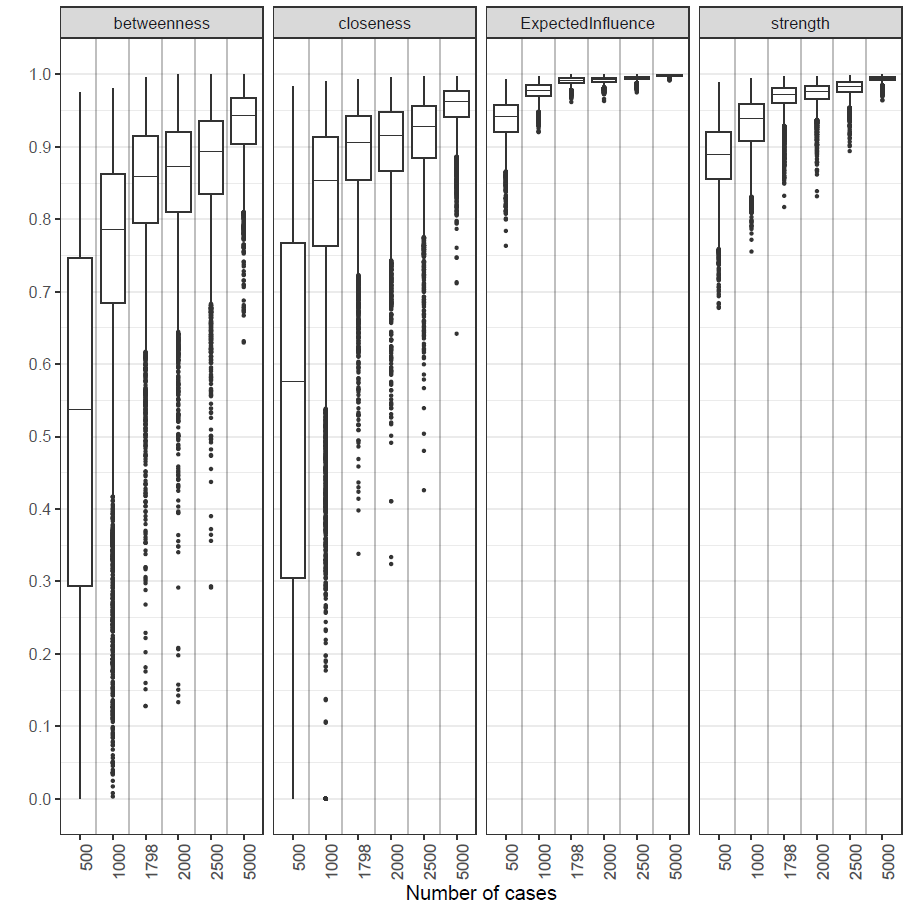


**References**

1. Ciudad-Fernández V, Zarco-Alpuente A, Escrivá-Martínez T, Herrero R, Baños R. How adolescents lose control over social networks: A process-based approach to problematic social network use. Addict Behav. 2024; 154:108003. doi:10.1016/j.addbeh.2024.108003.

2. Perales JC, King DL, Navas JF, Schimmenti A, Sescousse G, Starcevic V, et al. Learning to lose control: A process-based account of behavioral addiction. Neurosci Biobehav Rev. 2020; 108:771-780. doi:10.1016/j.neubiorev.2019.12.025.

3. Andrade B, García IG, Rial Boubeta A. Estudio sobre el impacto de la tecnología en la adolescencia. [Internet]. 2021 [cited 2024 Sep 4]. Available from: [www.unicef.es/infancia-tecnologia](http://www.unicef.es/infancia-tecnologia).

4. Pertegal MÁ, Oliva A, Rodríguez-Meirinhos A. Development and validation of the Scale of Motives for Using Social Networking Sites (SMU-SNS) for adolescents and youths. PLoS One. 2019; 14(12). doi:10.1371/journal.pone.0225781.

5. Romero Saletti SM, Van den Broucke S, Billieux J, Karila L, Kuss DJ, Rivera Espejo JM, et al. Development, psychometric validation, and cross-cultural comparison of the “Instagram Motives Questionnaire” (IMQ) and the “Instagram Uses and Patterns Questionnaire” (IUPQ). J Behav Addict. 2023; 12(1):105-127. doi:10.1556/2006.2022.00088.

6. Grant JS, Davis LL. Selection and use of content experts for instrument development. Res Nurs Health. 1997; 20(3):269-274. doi:10.1002/(sici)1098-240x(199706)20:3<269::aid-nur9>3.0.co;2-g.

7. Berk RA. Importance of expert judgment in content-related validity evidence. West J Nurs Res. 1990; 12(5):659-671. doi:10.1177/019394599001200507.

8. Yusoff MSB. ABC of content validation and content validity index calculation. Educ Med J. 2019; 11(2):49-54. doi:10.21315/eimj2019.11.2.6.

9. Davis LL. Instrument review: Getting the most from a panel of experts. Appl Nurs Res. 1992; 5:194-197. doi:10.1016/S0897-1897(05)80008-4.

10. Lynn MR. Determination and quantification of content validity. Nurs Res. 1986; 35(6):382-386.

11. Dalal DK, Carter NT, Lake CJ. Middle response scale options are inappropriate for ideal point scales. J Bus Psychol. 2014; 29(3):463-478. doi:10.1007/s10869-013-9326-5.

12. Padilla JL, Benítez I. Validity evidence based on response processes. Psicothema. 2014; 26:136-144. doi:10.7334/psicothema2013.259.

13. Boer M, van den Eijnden RJ, Finkenauer C, Boniel‐Nissim M, Marino C, Inchley J, et al. Cross‐national validation of the social media disorder scale: Findings from adolescents from 44 countries. Addiction. 2022; 117(3):784-795. doi:10.1111/add.15709.

14. Wang YA, Rhemtulla M. Power analysis for parameter estimation in structural equation modeling: A discussion and tutorial. Adv Methods Pract Psychol Sci. 2021; 4(1):2515245920918253. doi:10.1177/2515245920918253.

15. Golino HF, Epskamp S. Exploratory graph analysis: A new approach for estimating the number of dimensions in psychological research. PLoS One. 2017; 12(6). doi:10.1371/journal.pone.0174035.

16. Golino H, Shi D, Christensen AP, Garrido LE, Nieto MD, Sadana R, et al. Investigating the performance of exploratory graph analysis and traditional techniques to identify the number of latent factors: A simulation and tutorial. Psychol Methods. 2020; 25(3):292-320. doi:10.1037/met0000255.

17. Fruchterman TM, Reingold EM. Graph drawing by force-directed placement. Softw Pract Exp. 1991; 21(11):1129-1164. doi:10.1002/spe.4380211102.

19. Epskamp S, Borsboom D, Fried EI. Estimating psychological networks and their accuracy: A tutorial paper. Behav Res Methods. 2018; 50:195-212. doi:10.3758/s13428-017-0862-1.

20. Burger J, Isvoranu AM, Lunansky G, Haslbeck JMB, Epskamp S, Hoekstra RHA, et al. Reporting standards for psychological network analyses in cross-sectional data. Psychol Methods. 2023; 28(4):806-824. doi:10.1037/met0000471.

21. Bulteel K, Tuerlinckx F, Brose A, Ceulemans E. Using raw VAR regression coefficients to build networks can be misleading. Multivariate Behav Res. 2016; 51(2-3):330-344. doi:10.1080/00273171.2016.1150151.

22. Epskamp S. Network Psychometrics [Doctoral dissertation]. Amsterdam: University of Amsterdam; 2018.
